# The Genetic Network of Forkhead Gene Family in Development of Brown Planthoppers

**DOI:** 10.3390/biology10090867

**Published:** 2021-09-03

**Authors:** Hai-Yan Lin, Cheng-Qi Zhu, Hou-Hong Zhang, Zhi-Cheng Shen, Chuan-Xi Zhang, Yu-Xuan Ye

**Affiliations:** 1 The Rural Development Academy, Zhejiang University, Hangzhou 310058, China; 21616165@zju.edu.cn (H.-Y.L.); 22016223@zju.edu.cn (C.-Q.Z.); 2 Institute of Insect Sciences, Zhejiang University, Hangzhou 310058, China; 11716077@zju.edu.cn (H.-H.Z.); zcshen@zju.edu.cn (Z.-C.S.); chxzhang@zju.edu.cn (C.-X.Z.); 3 State Key Laboratory for Managing Biotic and Chemical Threats to the Quality and Safety of Agro-Products, Institute of Plant Virology, Ningbo University, Ningbo 315211, China

**Keywords:** forkhead-box, phenomics, RNA-seq, functional genetic network, brown planthopper

## Abstract

**Simple Summary:**

Forkhead (Fox) genes encode a family of transcription factors defined by a ‘winged helix’ DNA-binding domain and play important roles in regulating the expression of genes involved in cell growth, proliferation, differentiation and longevity. However, we still lack a comprehensive understanding of the Fox gene family in animals. Here, we take an integrated study, which combines genomics, transcriptomics and phenomics, to construct the Fox gene genetic network in the brown planthopper, *Nilaparvata lugens*, a major rice pest. We show that *FoxG*, *FoxQ*, *FoxA*, *FoxN1*, *FoxN2* and their potential target genes are indispensable for embryogenesis; *FoxC*, *FoxJ1* and *FoxP* have complementary effects on late embryogenesis; *FoxA*, *FoxNl* and *FoxQ* are pleiotropism and also essential for nymph molting; *FoxT* belongs to a novel insect-specific Fox subfamily; and *FoxL2* and *FoxO* are involved in the development of eggshells and wings, respectively. These findings may deepen our overall understanding of the regulatory function of the Fox gene family in insect growth and development, and thus ultimately stimulate the design and development of novel insecticides.

**Abstract:**

We identified 18 distinct Fox genes in the genome of the brown planthopper, *Nilaparvata lugens*, and further found a novel insect-specific subfamily that we temporarily named FoxT. A total of 16 genes were highly expressed in the eggs, while *NlFoxL2* and *NlFoxT* are female- and male-specific genes, respectively. Large scale RNAi and RNA-seq analyses were used to reveal the functions and potential targets of *NlFox*s. In the eggs, *NlFoxA*, *NlFoxN1* and *NlFoxN2* are indispensable to early embryogenesis by regulating different target genes; *NlFoxG* and *NlFoxQ* co-regulate *NlSix3* for brain development; and *NlFoxC, NlFoxJ1* and *NlFoxP* have complementary effects on late embryogenesis. Moreover, *NlFoxA*, *NlFoxNl* and *NlFoxQ* have pleiotropism. *Nl*FoxA and *Nl*FoxQ regulate the expression of *NlCHS1* and cuticular proteins, respectively, thereby participating in the formation of cuticles. *Nl*FoxN1, which regulates the expression of *NlKrt9* is involved in the formation of intermediate filament frameworks. Our previous studies have revealed that *NlFoxL2* and *NlFoxO* play important roles in chorion formation and wing polyphenism. Altogether, *N. lugens* Fox genes exhibit functional diversity in embryonic development and organogenesis. This comprehensive study combines genomics, transcriptomics and phenomics, thereby constructing a complex genetic network that spans the entire life cycle of the brown planthopper.

## 1. Introduction

The forkhead-box (Fox) genes encoded a large family of transcription factor (TF) characterized by a ‘winged-helix’ DNA-binding domain [1]. The first Fox protein was identified in the fruit fly *Drosophila melanogaster* [2], in which the conserved sequence of the DNA-binding domain is known as the Forkhead domain (FHD). This domain is very well conserved across the Fox family and across various eukaryote species; it extends about 100 amino acids in length [3].

Members of the Fox gene family have been identified in various species, ranging from yeast to human, and have evolved to acquire a specialized function in many key biological processes including fertility, metabolism and immunity [4,5,6]. Fox genes are commonly involved in early embryogenesis [7]. During embryogenesis, Fox genes are required for the organogenesis of multiple systems such as the liver, lungs, kidney and central nervous system [8,9,10].

There are 50 Fox genes in the human genome and 44 in the mouse, each of which can be divided into 19 subfamilies (FoxA to FoxS) based on the sequence homology of the FHD [6,11]. The FoxR and FoxS subfamilies are specific to the vertebrate [12]. It was not possible to identify the FoxE, FoxH, FoxI and FoxM subfamilies in *D. melanogaster*, *Anopheles gambiae* or *Caenorhabditis elegans*, suggesting either a considerable loss in ecdysozoans or the evolution of these subclasses in the deuterostome lineage [13]. The fruit fly has 18 Fox genes and 13 subfamilies, while *Bombyx mori* has 17 Fox genes and 13 subfamilies [13,14]. There are 18 Fox genes in the *Aedes aegypti* genome, and the knockdown of *FoxN1*, *FoxN2*, *FoxL2* or *FoxO* has a negative effect on reproduction [15].

The brown planthopper (BPH), *Nilaparvata lugens* (Stål) (Hemiptera: Delphacidae), is one of the most destructive insect pests of rice crops; the feeding activity of the BPH causes significant losses in rice yields annually in Asia by directly sucking up the phloem sap and transmitting plant viruses. The completion of the genome sequencing of the BPH combined with a significant amount of transcriptome sequencing data provided us with an opportunity to conduct a global analysis of the gene families in this important pest insect [16]. Thus far, most studies have focused on an individual gene function, and thus, lack a comprehensive understanding of the Fox gene family.

In this study, we identify all the genes of the Fox gene family in the *N. lugens* genome and the annotated cDNA sequences. The gene architectures, phylogenetic relationships, expression patterns and RNA interference (RNAi) results are analyzed to elucidate the functions of the Fox genes with the context of BPH growth, development and reproduction. Moreover, RNA-seq is used to further explore the differentially expressed genes (DEGs) of these Fox genes. As the expression levels of the target genes are highly correlated with the TF expression levels [17], the expression feature correlation matrix was introduced to further screen out the potential target genes from the DEGs. Ultimately, we constructed a coordinated genetic regulatory network of the Fox gene family throughout the life cycle of the BPH.

## 2. Materials and Methods

### 2.1. Gene Identification

*Nilaparvata lugens* genomic (GenBank accession numbers: JADOXM000000000) and transcriptomic databases [16] were screened for Forkhead genes against the amino acid sequences from *Homo sapiens*, *Mus musculus*, *Strongylocentrotus purpuratus*, *Drosophila melanogaster*, *Bombyx mori* and *Aedes aegypti*, which were obtained from GenBank. The full-length of the cDNA sequences were obtained from transcriptomic databases, and then cloned using ClonExpress II One Step Cloning Kit (Vazyme, Nanjing, China). The primers (synthesized by Sunya, Shanghai, China) used here can be found in Supplementary Materials, Table S1.

### 2.2. Sequence Analysis

Multiple sequence alignments were performed using the ClustalX program [18]. The ORF prediction was performed on the Softberry website (http://www.softberry.com/ (accessed on 1 March 2021)). The SMART program (http://smart.embl-heidelberg.de/ (accessed on 1 March 2021)) was employed for the identification of modular domains. The phylogenetic trees were constructed via the maximum likelihood method using the MEGA7.0 program [19]. Homologous relationships were determined using a bootstrap analysis of 1000 replications, as each legend described.

### 2.3. Real-Time Quantitative PCR (qRT-PCR) Analysis

Total RNA from whole insects at various developmental stages or tissue samples was isolated using a TRIzol Total RNA Isolation Kit (Takara, Kyoto, Japan). First-strand cDNA was synthesized using a HiScript II 1st Strand cDNA Synthesis Kit with gDNA wiper (Vazyme Biotech Co., Ltd., Nanjing, China) using 0.5 μg of total RNA template in a 10-microliter reaction, following the manufacturer’s protocol. QRT-PCR was conducted using pairs of gene-specific primers with high efficiencies, which were designed using the Primer Premier 6 program (Supplementary Materials, Table S1). The qRT-PCR reactions (20 μL each) contained 2 μL of cDNA diluted 10-fold, 0.6 μL of each primer and 10 μL of ChamQ SYBR Color qRT-PCR Master Mix (Vazyme Biotech Co., Ltd.). The reactions were run in a Bio-Rad Real-time PCR system (Bio-Rad, Hercules, CA, USA). The *N. lugens* housekeeping gene for 18S ribosomal RNA (*Nl18S*) (GenBank accession number JN662398.1) was used as an internal control. The qRT-PCR program consisted of an initial denaturation step at 95 °C for 30 s, followed by 40 cycles at 95 °C for 5 s and 60 °C for 30 s. Three biological replications were performed for each sample. A relative quantitative method (^ΔΔ^C_t_) [20] was applied to evaluate the variation in expression among the samples.

### 2.4. Expression Pattern Analysis

Developmental samples were collected from different stages of BPHs, including 8 egg samples, 30 nymph samples, 7 female adult samples and 4 male adult samples. Similarly, various tissue samples, including integument, gut, fat body, salivary gland, testis and ovary were dissected from random adults 48–72 h after adult emergence. Three biological replications were performed for each developmental and tissue sample. Spatial and temporal expression patterns of *N. lugens* Fox genes were investigated via qRT-PCR.

### 2.5. RNAi Effects

The double-stranded RNA (dsRNA) was synthesized from the purified DNA templates, which were prepared via the amplification of RT-PCR, which was accomplished using a T7 High Yield RNA Transcription Kit (Vazyme, Nanjing, China). A unique region of each gene was chosen as a template for dsRNA synthesis. The primers used for the dsRNA synthesis can be found in Supplementary Materials, Table S1. The dsRNA for *GFP* was used as a negative control for the nonspecific effects of dsRNA. Microinjection of planthoppers with dsRNA was carried out according to a previously reported method [21]. Briefly, dsRNA was injected into the membrane between meso- and meta-thoracic legs using a microinjection needle under a stereomicroscope. About 150 insects were used for each gene treatment, and each treatment was conducted in triplicate. Each insect was injected with 10 μL of dsRNA, at a concentration of 5 μg/μL. Samples were collected from a set of 6–10 insects to evaluate the RNAi effects of each gene. A second, non-overlapping region was selected for dsRNA synthesis to overcome possible off-target effects. The regions were designed to have no other similar sequences in the genome (Supplementary Materials, Figure S1).

### 2.6. RNA-Seq

The newly emerged female adults were injected with dsRNA, paired with normal male adults and kept for three days on fresh rice seedlings until they reached sexual maturity. Approximately 500 eggs newly laid on rice stems (for *FoxA/N2*) and 500 eggs 48 h after being laid (for *FoxG/Q*) were carefully dissected for total RNA extraction. Thirty individuals, 48 h after injecting dsRNA in the fourth instar nymphs, were homogenized for total RNA extraction (for *Fox*A/Q). The dsRNA for *GFP* was used as a negative control for the nonspecific effects of the dsRNA. Each treatment involved three sets of biological replicates. The cDNA library preparation and Illumina sequencing were performed by Annoroad (Beijing, China). The clean reads were aligned to the *N. lugens* reference genome using the alignment software HISAT2 [22]. Aligned reads were counted by feature counts [23]. The differentially expressed genes (DEGs) were identified by DESeq2, satisfying the following conditions: false discovery rate (FDR) < 0.05 and absolute value of the log2 ratio > 2 [24].

## 3. Results

### 3.1. Identification and Sequence Analyses of Forkhead Genes in the Brown Planthopper

The BLAST searches of the *N. lugens* genomic and transcriptomic databases identified 18 genes encoding Forkhead transcription factors. The cDNAs of the 18 genes were amplified, cloned and sequenced (Supplementary Materials, Figure S1). The predicted amino acid sequences were further analyzed to identify the key features. A domain architecture analysis revealed that all the members of the *N. lugens* Fox proteins contain Forkhead domains (Supplementary Materials, Figure S2a). The multiple sequence alignments performed on the 18 FHDs revealed that the amino acid sequences of the *N. lugens* FHD are highly conserved, especially the N-terminal amino acids (Supplementary Materials, Figure S2b). The FoxO superfamily is the most divergent subfamily of the Fox family due to its unique five amino-acid (GDSNS) insertion immediately prior to helix H3 [25]. *Nl*FoxO contains this unique sequence, which is involved in sequence-specific interaction with DNA-binding sites.

**Figure 1:**
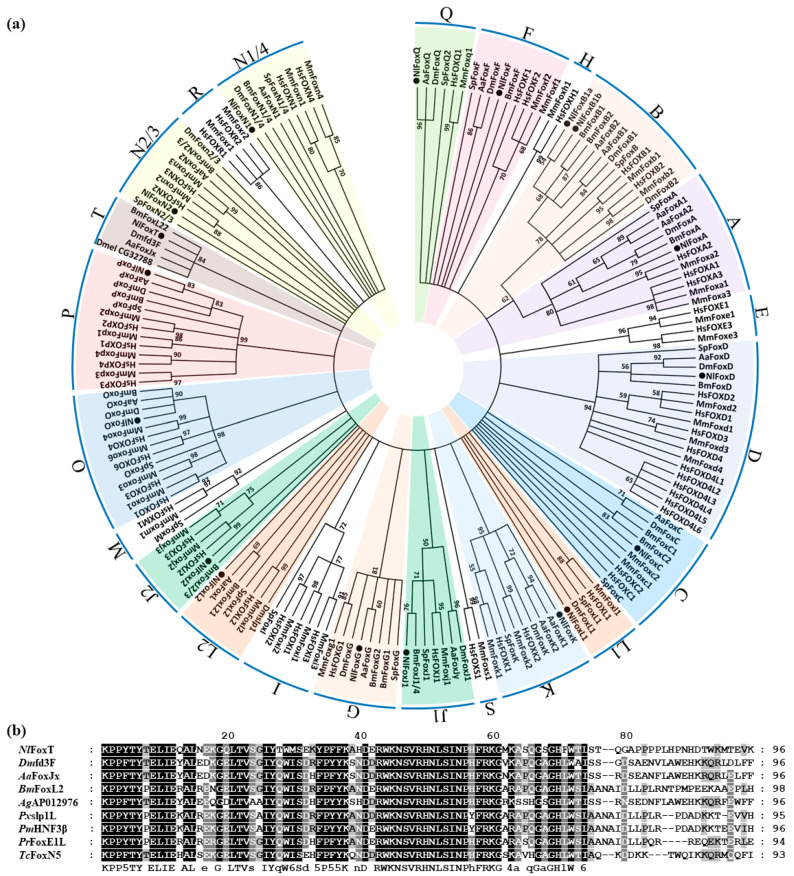
Several insect Fox genes clustered to form an insect-specific branch. (**a**) Phylogenetic analysis of Fox proteins. The tree was constructed using MEGA7 software with the maximum likelihood method. A bootstrap analysis of 1000 replications were conducted, and bootstrap values (%) are presented in the cladogram. For condensing the tree, 50% cut-off values were used. Different subfamilies in *N. lugens* were painted in different colors. (**b**) Alignment of amino acid sequences of FHD from insect-specific Fox genes. Nl: *Nilaparvata lugens*, Hs: *Homo sapiens*, Mm: *Mus musculus*, Sp: *Strongylocentrotus purpuratus*, Dm: *Drosophila melanogaster*, Bm: *Bombyx mori*, Aa: *Aedes aegypti*, Ag: *Anopheles gambiae*, Px: *Papilio xuthus*, Pm: *Papilio machaon*, Pr: *Pieris rapae*, Tc: *Tribolium castaneum*.

To examine the classification of the Fox genes in *N. lugens*, we collected the FHD sequences of the gene families from seven different species to construct a phylogenetic tree, including *N. lugens*, *Homo sapiens*, *Mus musculus*, *Strongylocentrotus purpuratus*, *D. melanogaster*, *Bombyx mori* and *Aedes aegypti*. The well-studied Fox gene families in the two mammals contributed to the classification accuracy. The results indicate that 17 of the 18 *N. lugens* Fox proteins were assigned to 13 normal subfamilies. According to all analyses, FoxA, FoxB, FoxD, FoxG, FoxK, FoxO and FoxP are well-supported, monophyletic subfamilies. The rest of the subfamilies exhibited phylogenetic diversity after the 50% cut-off values for condensing the tree. FoxJ, FoxL and FoxN were further divided into two assemblages. We were unable to identify the orthologs of Fox subclasses E, H, I, M, R and S in insects (Figure 1a).

In addition to these well-defined Fox genes, all the remaining insect Fox genes were not found to belong to any one of the known subfamilies, and instead clustered to form an insect-specific Fox gene branch. The existence of this branch is strongly supported by a high bootstrap value of 84% (Figure 1a). We temporarily named this branch FoxT, according to common naming convention [1]. The multiple sequence alignment of amino acid sequences in the FHD region of FoxT from different insects revealed that the N-terminus of these protein sequences is highly conserved (Figure 1b).

### 3.2. Temporal and Spatial Expression Patterns

The spatial and temporal expression patterns of different Fox gene transcripts were determined using a qRT-PCR.

**Figure 2:**
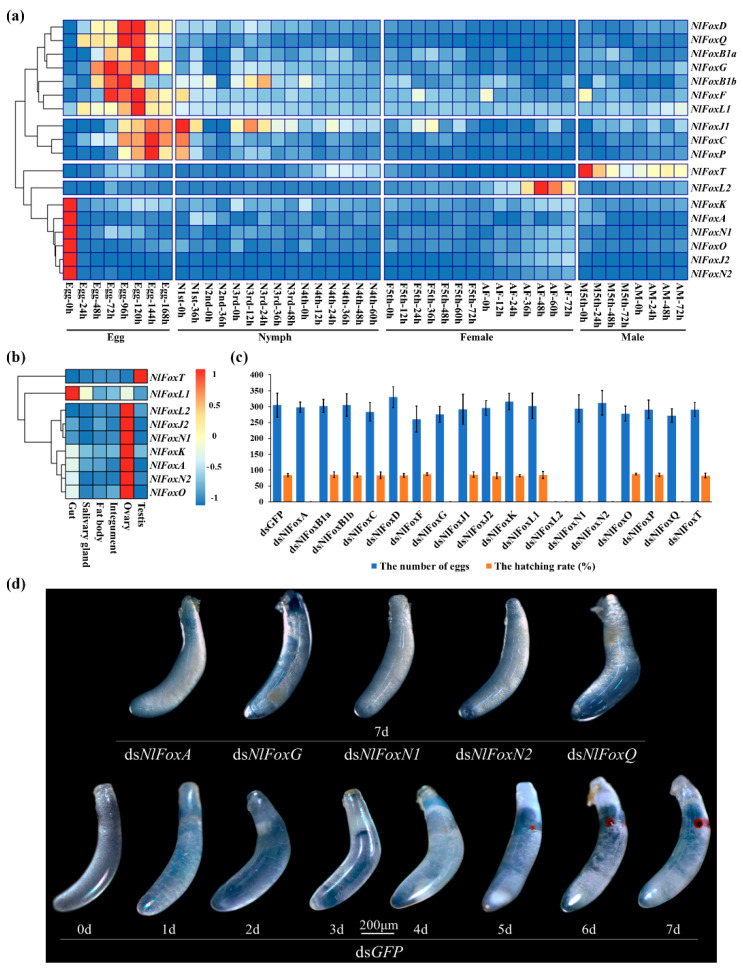
*N. lugens* Fox genes are highly expressed in eggs and play indispensable roles in fecundity and hatchability. (**a**) Expression patterns of Fox genes in different development stages. N: nymph; AF: adult female; M: male; AM: adult male. (**b**) Expression patterns of Fox genes in different tissues. (**c**) Effect of RNAi on fecundity and hatchability. DsRNA except for *NlFoxT* (50 ng per insect; *n* = 100) was injected into newly emerged female adults (within two hours). DsRNA for *NlFoxT* (50 ng per insect; *n* = 100) was injected into newly emerged male adults (within two hours). Mean ± standard error of the mean (s.e.m.) from three experiments. (**d**) The lethal phenotypes of eggs injected with dsRNA for *N. lugens* Fox genes. DsRNA for *NlFoxA*, *NlFoxG*, *NlFoxN1*, *NlFoxN2* and *NlFoxQ* (50 ng per insect; *n* = 100) was injected into newly emerged female adults (within two hours). Ds*GFP* was injected as a negative control for the nonspecific effects of dsRNA.

To explore the developmental expression patterns, BPHs were sampled at different time points based on embryogenesis, growth, development and reproduction (Figure 2a). The tissue samples used for the tissue-specific expression patterns were extracted from random adults (Figure 2b). Our analysis found that 18 *N. lugens* Fox genes could be divided into five groups for the developmental expression patterns. Of the 18 *N. lugens* Fox genes, 16 were highly expressed in the egg stage. Thus, these genes might play an important role in the embryonic development of the fertilized eggs. *NlFoxA*, *NlFoxJ2*, *NlFoxK*, *NlFoxN1*, *NlFoxN2* and *NlFoxO* were also found to be highly expressed in the newly laid eggs (early stage) and in the ovaries, suggesting that the eggs had begun to express these genes in the maternal. *NlFoxB1a*, *NlFoxB1b*, *NlFoxD*, *NlFoxF*, *NlFoxG, NlFoxL1* and *NlFoxQ* had similar developmental expression patterns, with transcripts peaking in the middle stage of the eggs. *NlFoxC*, *NlFoxJ1* and *NlFoxP* were highly expressed in the late stage of the eggs to the early stage of the nymphs. *NlFoxT* was hardly expressed in the eggs, young nymphs, fifth instar female nymphs or female adults, but was highly expressed in the fifth instar male nymphs and male adults, indicating that *NlFoxT* is a male-specific gene. Furthermore, *NlFoxT* exhibits distinct tissue specificity and is specifically expressed in the testis. *NlFoxL2* is highly expressed in the ovaries of the females. Other *N. lugens* Fox genes are rarely expressed in the nymphs and adults, and we were thus unable to detect their tissue-specific expression patterns.

### 3.3. NlFoxG and NlFoxN2 Are Indispensable for Embryogenesis

To determine the functions of the *N. lugens* Fox genes, we conducted RNAi experiments. To avoid off-target effects, the RNAi experiments were replicated by choosing two non-overlapping regions as targets. The qRT-PCR analysis revealed that each dsRNA efficiently suppressed the transcript levels of their target genes (Supplementary Materials, Figure S3). Most *N. lugens* Fox genes, except *NlFoxL2* and *NlFoxT,* were highly expressed in the egg stage. Since parental RNAi has been found to occur in BPHs [26], we conducted the RNAi experiments on newly emerged female adults, before their ovaries had the chance to mature, to observe the phenotypes of egg production and embryos from the next generation.

The knockdown of *NlFoxG* or *NlFoxN2* efficiently suppresses the hatching rate but does not affect the oviposition number (Figure 2c). These eggs cannot develop normally. The red eyespots (compound eye buds) that should appear about 4 days after egg production did not appear, without exception, indicating that the embryo development is terminated before the stage of embryonic movement (Figure 2d). RNA-seq has become a powerful tool to investigate transcriptome profiling using deep-sequencing technologies [27]. We sought to use RNA-seq to reveal the potential target genes of *NlFoxG* and *Nl*FoxN2 in the eggs.

In total, 50 DEGs were found after the knockdown of *NlFoxN2* in the eggs, including 21 upregulated and 29 downregulated genes (Supplementary Materials, Table S2). A qRT-PCR confirmed that seven genes were downregulated by *NlFoxN2*. The RNAi results further indicate that four of the seven genes are indispensable to embryogenesis (Supplementary Materials, Table S3), suggesting they are the potential target genes of *NlFoxN2,* and therefore, account for the lethal effect of *NlFoxN2* knockdown in early egg stages.

For *NlFoxG*, there were 214 upregulated and 84 downregulated genes (Supplementary Materials, Table S4). One of the 84 downregulated genes was *Six3* (sine oculis homeobox homolog 3); qRT-PCR confirmed that *NlSix3* is downregulated by *NlFoxG* (Supplementary Materials, Figure S4a). The eggs were unable to hatch due to the knockdown of *NlSix3* (Supplementary Materials, Figure S5). Moreover, the eyespots were much smaller than the control group 7 days after egg production (Supplementary Materials, Figure S6).

### 3.4. Pleiotropic Functions of Fox Genes (A/N1/Q) and Their Potential Targets in the Brown Planthopper

Our previous study has revealed that *NlFoxN1* exhibits pleiotropism during embryogenesis and nymph molting [28]. The results of the RNAi experiments indicate that *NlFoxA* and *NlFoxQ* have a similar pleiotropism: they not only affect the egg hatching rate, but also play an important role in molting.

**Figure 3:**
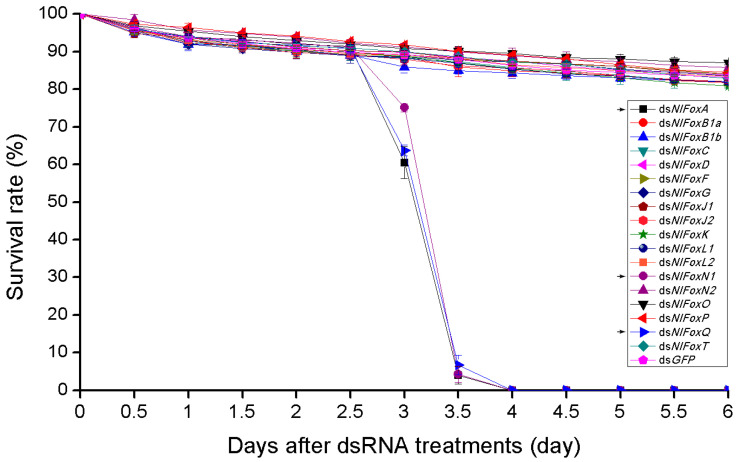
The survival rate after RNAi in nymphs. DsRNA (50 ng per insect; *n* = 100) was injected into BPHs at the very beginning of the fifth instar. The survival rate started calculating every 12 h after injection. Mean ± s.e.m. from three experiments.

**Figure 4:**
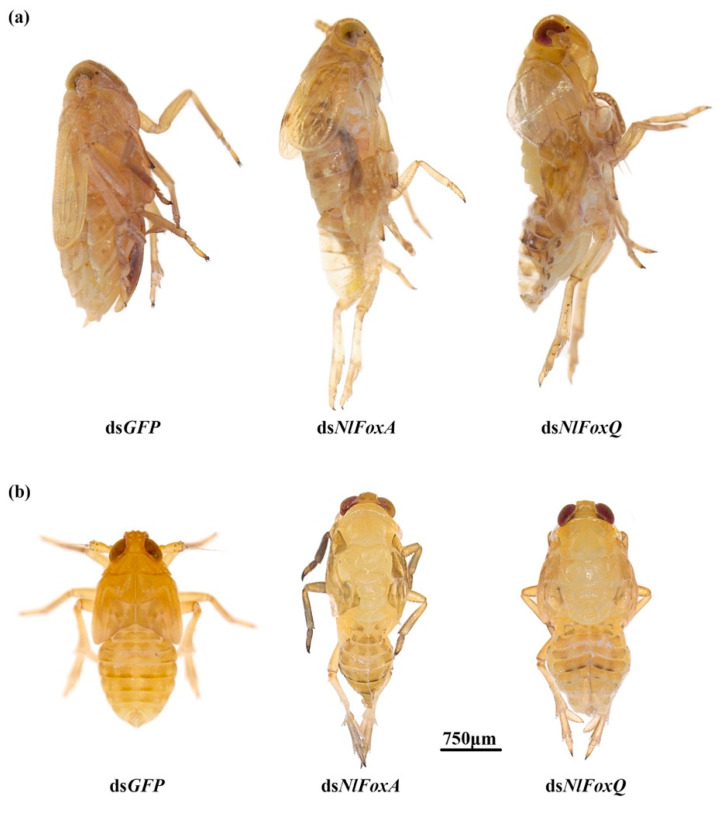
The lethal phenotypes of insects injected with dsRNA for *N. lugens* Fox genes. (**a**) DsRNA for *NlFoxA* or *NlFoxQ* (50 ng per insect; *n* = 100) was injected into BPHs at the very beginning of the fifth instar; (**b**) dsRNA for *NlFoxA*, *NlFoxN1* or *NlFoxQ* (50 ng per insect; *n* = 100) was injected into BPHs at the very beginning of the fourth instar. Ds*GFP* was injected as a negative control for the nonspecific effects of dsRNA.

The depletion of *NlFoxA* or *NlFoxQ* in the eggs prevented the development of the eggs, resulting in a hatching rate of zero (Figure 2c). An injection of dsRNA for *NlFoxA* or *NlFoxQ* in the early fifth instar nymphs led to a lethal phenotype, after which the mortality rate reached almost 100% (Figure 3). All the dead BPHs exhibited the same phenotype. The dead BPHs failed to shed their old cuticles and died quickly during nymph-to-adult molting (Figure 4a). The same phenotype was found between the 4th-to-5th nymph molting after injecting *NlFoxA* or *NlFoxQ* dsRNA into the early fourth instar nymphs (Figure 4b).

The pleiotropic roles of Fox proteins during the embryonic development and homeostasis of adult tissues are supported by the ability to coordinate the temporal and spatial gene expression of their target genes [5]. *NlFoxN1* regulates 10 potential target genes to initiate embryogenesis in the eggs and regulates keratin genes to maintain the homeostasis of nymph cuticles [28]. To investigate the potential target genes of *NlFoxA* and *NlFoxQ* in different stages, we used RNA-seq to profile the transcriptome after RNAi in eggs and nymphs, respectively.

In total, 93 genes were found to be significantly differentially expressed after the knockdown of *NlFoxA* in the fourth instar nymphs, including 10 upregulated genes and 83 downregulated genes (Supplementary Materials, Table S5). Among these genes, a gene encoding chitin synthase 1 (CHS1) particularly caught our attention, as CHS is required for chitin formation in insect cuticles and other tissues [29]. Silencing *NlCHS1* resulted in a high mortality rate during the molting period [30]. The qRT-PCR result confirms that *NlCHS1* is regulated by *Nl*FoxA (Supplementary Materials, Figure S4b).

In the eggs, there were 8 upregulated genes and 28 downregulated genes after the knockdown of *NlFoxA* (Supplementary Materials, Table S6). Among these, three genes were verified using a qRT-PCR. The RNAi results show that three genes are indispensable to embryogenesis (Supplementary Materials, Table S7).

In the fourth instar nymphs, 97 differentially expressed genes (72 upregulated and 25 downregulated) between *NlFoxQ*-RNAi and control treatments were identified (Supplementary Materials, Table S8). One cuticular protein gene was reflected in our eyes: Cpr56 (cuticular proteins with the R&R consensus 56). Previous studies have confirmed that RNAi against *NlCpr56* resulted in a lethal phenotype [26]. The qRT-PCR results confirm that *NlCpr56* is regulated by *Nl*FoxQ (Supplementary Materials, Figure S4c).

There are 1904 upregulated genes and 843 downregulated genes in the *NlFoxQ*-RNAi eggs (Supplementary Materials, Table S9). One of them is *NlSix3*. The qRT-PCR confirmed that *NlSix3* is downregulated by *NlFoxQ* (Supplementary Materials, Figure S4a).

### 3.5. Complementary Functions of NlFoxC, NlFoxJ1 and NlFoxP in the Late Embryonic Stage

Except in *NlFoxA*, *NlFoxG*, *NlFoxN1*, *NlFoxN2* and *NlFoxQ*, an injection of dsRNA targeting any single gene of the remaining *N. lugens* Fox genes does not obviously affect embryonic development. Combined RNAi experiments were performed to study the compensatory effects of *N. lugens* Fox genes during embryogenesis. Three groups were divided according to their expression patterns (Group—Early: *NlFoxJ2*, *NlFoxK* and *NlFoxO*; Group—Middle: *NlFoxB1a*, *NlFoxB1b*, *NlFoxD*, *NlFoxF* and *NlFoxL1*; Group—Late: *NlFoxC*, *NlFoxJ1* and *NlFoxP*). Surprisingly, the knockdown of *Group—Late* led to a hatching rate of 0% (Supplementary Materials, Figure S5). The eyespot was dark red, and its diameter was approximately one quarter of the egg, suggesting that the egg had developed to the late stage (Supplementary Materials, Figure S6). These eggs were unable to hatch normally. This result suggests that Fox genes might have compensatory effects on each other.

## 4. Discussion

Early stage of eggs: Six *N. lugens* Fox genes were highly expressed in the very beginning of the eggs and three of them (A/N1/N2) were indispensable to embryonic development. The representatives of the FoxA subfamily are pioneer factors and are involved in the regulation of cell differentiation in the early stages of embryonic development by interacting with condensed chromatin and making the regulated genes available for activation [31]. *Nl*FoxA was likely to act similarly to the mammalian FOXA subfamily in embryogenesis, being used as a pioneer factor. The similarities in the FHD sequences, expression patterns and RNAi phenotypes among *NlFoxN1*, *NlFoxN2* and *NlFoxA* suggest that *Nl*FoxN1 and *Nl*FoxN2 had a similar pioneer activity with *Nl*FoxA in the eggs. Such activity may explain the broad range of functions regulated by these genes in the newly laid eggs.

Middle stage of eggs: Seven *N. lugens* Fox genes are highly expressed in the middle stage of the eggs and two of them (G/Q) are indispensable to embryonic development. Foxg1 plays a non-redundant role in vertebrate brain development, with its unique expression in the embryonic telencephalon [32]. The expression pattern of the *FoxG* homologue in *Drosophila* is found in the embryonic head region [33]. In *T. castaneum*, *FoxQ2* co-expressed with *Six3* was required for central brain patterning [34]. Moreover, *TcSix3* is required for the embryonic formation of median brain development, which is very likely conserved among bilaterians [35]. In *N. lugens*, *Six3* is indispensable to embryogenesis. RNA-seq analysis reveals that *NlSix3* is downregulated both by *NlFoxG*-RNAi and *NlFoxQ*-RNAi, which was confirmed using a qRT-PCR. We hypothesize that *NlFoxG* and *NlFoxQ* affect embryonic development by regulating brain development.

Late stage of eggs: *NlFoxC*, *NlFoxJ1* and *NlFoxP* are highly expressed in the late stage of the eggs and continue to the early stage of the nymphs. The knockdown of these genes alone does not result in any abnormal phenotypes. They affect embryonic development through complementary effects. However, we have yet to understand how Fox genes interact with one another in complementary ways.

Nymphs: A previous study mentioned that *NlFoxA* plays an important role in the regulation of fecundity and the development of ovaries in BPHs [36]. In *spodoptera exigua*, suppressing the expression of FoxA interrupts expression levels of *SeCHSA* and *SeCHSB*, then disrupts the chitin biosynthesis pathway [37]. The RNA-seq results demonstrate that *Nl*FoxA participates in chitin synthesis by regulating *NlCHS1*. It is confirmed that the missense mutations in Foxq1 affect hair development in mice [38]. The cuticle is a major hair shaft compartment [39]. Our experiments have found that *Nl*FoxQ participates in the molting process by regulating a cuticular protein, *NlCpr56*. Insect cuticles are a complex composite material, made of chitin filaments embedded in cuticular proteins [40]. It provides structural and mechanical support by serving functionally as both the skin and the skeleton [41]. Thus, it has been proposed that *Nl*FoxA and *Nl*FoxQ regulate the formation of cuticles in *N. lugens* by regulating the chitin and cuticular proteins, respectively. *Nl*FoxN1 participates in the formation of the IF framework by regulating the expression of *NlKrt9* (type I cytoskeletal keratin 9)*,* thereby influencing the molting process. Our previous study has found that *Nl*FoxO plays an important role as a wing morph switch in the regulation of the wing polyphenism in the BPH [21].

Males and Females: *NlFoxL2* and *NlFoxT* are gender-specific genes. Our previous study has revealed that *Nl*FoxL2 activates *NlFcp3C* to regulate chorion formation in the female ovaries [42]. *NlFoxT* exhibits distinct sex specificity and tissue specificity and is mainly expressed in the testis of the males. Members of this subfamily generally exhibit this specificity. This discovery might provide a valuable clue for the classification and functional research of the Fox gene family. However, we did not discover any visible abnormalities in males after the knockdown of *NlFoxT*.

## 5. Conclusions

We identified a total of 18 Fox genes from the genomic and transcriptomic databases of the BPH and cloned their cDNA sequences. Among the 18 *N. lugens* Fox proteins, 17 belong to the 13 known subfamilies, while the other one clustered with insect Fox proteins to form an insect-specific Fox protein branch. The results of the functional study indicate that the *N. lugens* Fox genes had functional diversity in embryonic development and organogenesis. At different stages, different members of *N. lugens* Fox genes regulate their downstream genes to serve different functions, thus constructing a coordinated genetic network spanning the entire life cycle.

## Data Availability

Not applicable.

## Supplementary Materials

The following are available online at https://www.mdpi.com/article/10.3390/biology10090867/s1. Figure S1: The cDNA sequences of 18 BPH Fox genes, Figure S2: Sequence analyses of Forkhead genes in *N. luge*, Figure S3: RNAi efficiency by was determined by qRT-PCR, Figure S4: (a) Knockdown of *NlFoxG* or *NlFoxQ* both decreased expression levels of *NlSix3* in the eggs (b) Knockdown of *NlFoxA* decreased expression levels of *NlCHS1* in the nymphs (c) Knockdown of *NlFoxQ* decreased expression levels of cuticular protein genes in the nymphs, Figure S5: Knockdown of *Group-Late* or *NlSix3* prevented the hatchability, Figure S6: Lethal phenotypes of eggs injected with dsRNA for *Group-Late* or *NlSix3*. Table S1: Primers used in this work, Table S2: The differentially expressed genes after knockdown of *NlFoxN2* in the eggs., Table S3: Top 7 differentially down-regulated genes after knockdown of *NlFoxN2* in the eggs., Table S4: The differentially expressed genes after knockdown of *NlFoxG* in the eggs., Table S5: The differentially expressed genes after knockdown of *NlFoxA* in the nymphs, Table S6: The differentially expressed genes after knockdown of *NlFoxA* in the eggs., Table S7: Top 3 differentially down-regulated genes after knockdown of *NlFoxA* in the eggs, Table S8: The differentially expressed genes after knockdown of *NlFoxQ* in the nymphs, Table S9: The differentially expressed genes after knockdown of *NlFoxQ* in the eggs.
