# Supplementary material for: The Genetic Network of Forkhead Gene Family in Development of Brown Planthoppers
Source: Biology (Basel). 2021 Sep 3;10(9):867. doi: 10.3390/biology10090867 (PMC8469257; doi:10.3390/biology10090867)
Supplement: Supplementary file 1 [file biology-10-00867-s001.zip › biology-1352669-supplementary.pdf]

biology MDPI

Supplementary Materilas: Figures S1–S6

>*NlFoxA*¶

CTGTTGCGGAGGTTATGTTGTGTTGTATAGACTGATTGATAGTGATAGTTTGAAGAATGACGATGTTGTCGCAAAAGT
TGTACGCGGATTCGGTGAGCAGTGCGATGGGCACGAACGCGCCCGGCTACGGAATGAACTCCATGATGAACTCGTGC
TCACCTCAGGGTGCCTCAGGATTCAACATGCTACAAGGTTCAGCCGCTATGGCCGGCCTCAACGGCAACTGTATGGG
CGGGGGGACCATGGGCTATGGGGCACAGGGAGTAGGCTCGTCGTGTATGGGGCCCTACGGCGCGGCCCCACTCACCT
CCAGGGCGGACGTCACCCCGTCGGCGCCTCCCGGTGAAGCCGCGTCACCTTCCGCTCTGCAGAGAGCTCGTGCCGAC
AAATCCTACCGGAGGAGTTACACGCACGCTAAACCACCCTACTCGTACATTTCGCTCATCACGATGGCCATCCAGAA
TTCTCCCACCCGCATGCTCACACTATCAGAGATCTATCAGTTCATAATGGACCTGTTTCCGTTCTACAGGCAAAACCA
GCTGCGGTGGCAGAACTCGATTAGGCATTCGTTGAGTTTCAATGACTGTTTTGTGAAGGTGCCGCGAACGCCGGACAA
GCCGGGCAAAGGCAGCTTCTGGACGCTGCATCCCGAGTCGGGCAACATGTTCGAGAACGGCTGCTACCTGCGGAGG
CAAAAGCGGTTCAAGGACGAGAAGAAGGAGGCTGTGAGACAGACGCACAAGAGTGCGGTGAGTCCGTCGCACCAC
GGACACCAAACACCGCACACGCAGCATCTGCACGACGAGAAGCTCAAGGAGAAGGTGGAGGACGTGGTTCTGCATG
CGTCACACGTTGACCTGTGCTCGGCTGCCTCACAGCTGCACCAGCGGCAACAACAGTCGCATCACCAGCAACAACAA
CAACAGCACCAGGACGAGATGGTCGCTGCCATGGTGGGACGTTGTCATCCGCACCTGGCCGCCTCCCTGCAGGTGCA
GCCCGACACGCACTACCCTCACTCGCACCACCACCTGAAGCAGGAGTACCCCGCATCCAGCCACCCGTTCTCCATCA
CCCGGCTGCTGCCCGCCGAGAGCAAGACAGACATCAAAATGTATGACATGTCCCAGTATGGCTACAACGACTACTAC
CAGAGCTCGCTCTACCACCCTTCGGCCGGCACCACCGCCTTGTGACAGTATCCGCTCGCCTAAG¶

>*NlFoxB1a*¶

CGGATGATAGTGTGATTAGTTCTCGATAAGCATTTCATGGAACAGTTTTTCAGGGAGATTGGAAGTTAGTTTTTCTGAT
GATAGTGTGACAATTTTCCGACAAACATGCCACGACCCTGTCGTGAATCGTACGGCGATCAGAAGCCTCCCTATTCCT
ACATTTCGCTGACCGCTATGGCGATATGGAGCTCGCCCGACAAAATGCTACCTCTCTCAGACATTTATCGTTTTATCAG
TGATCGATTTCCATACTACAGAAGAAACACACAACGCTGGCAAAATTCTCTGAGACATAATTTGTCGTTCAATGATTG
TTTCGTGAAGATACCTCGGAGACCTGATAGGCCAGGGAAAGGCGCTTACTGGGCGTTACACCCGGCAGCGTTAGACA
TGTTTGAGAATGGATCGTTGTTAAGGAGACGTAAGAGGTTCAAACTGATGAAATCTGACAAGGATAGATTGGAGAAC
GAGTTGGCAGCGCTGGCGAACATGAATCGGTTCCTGTTCGCCCCTCAACAGCCGCCTCCAGCCGCCCCACAACCCTC
GCCCCCGGAGATGGTGCTTTCGGAGCCGCCTCTCACTAGTTCTGAGCCGCCTCCGAGACTTAAGCGCGACTTCACGAT
TGCCAGCCTGATTTCGCCTGATAGACCAAGGCCTGCATGCTCACCACCTCCGGCGATACATGTGGTGCCCTGCTGTCC
GCCGCCTCCACCGCCTCTAGTCCTTCTGCCGCCTCCAAGTCACGTGGCTCTCCCGCCGCCCCCGGGGTACTTCCATCAC
CACCACCATCTACCGCCTCCCAGTCACCATGTGTTCAGGTTTGGGCCGACGTTGAGCGCCGTTTGACCTCCGGAAACC
TTCCAGAAAATCTGGTAAAACAGTGATAATTATTTTGTATTGTTAGTAAAGTTCCATTAGTCAGTTAAGTTAGCGTCTC
AAGC¶

>*NlFoxB1b*¶

GGAAACCTTTAATCCCTGAAAGGGACAATGCCACGACCCTGTCGTGAGTCGTACGGTGACCAAAAACCTCCCTATTC
CTACATTTCCCTGACCGCTATGGCGATATGGAGTTCACCCGAAAAAATGCTCCCCTTATCAGACATTTATCGGTTTATC
AGTGATCGATTCCCATACTACAGGAGAAACACTCAACGGTGGCAAAATTCTTTGAGACATAATTTGTCGTTCAATGAT
TGTTTTATTAAAATACCTCGCAGACCTGATAGACCAGGGAAAGGCGCTTACTGGGCGCTACACCCTGCAGCGTTAGA
TATGTTTGAAAACGGGTCGTTCCTCAGGAGGCGGAAAAGGTTTAAATTGCCGAAACTGGAGAATTTGGTGAAGAATT
CTTCATCTGCCAGCCCCAAGGCCCCCGAGGGACCCTCTTCAAGCCCCCCTGTGACTACCGAAGTTCCTAAGAGCGCC
GCCCCCAAGCCGTTCAGTATCGACAGCATAATTTCGCCGTCACCGCCCCCAGCGAGCAATTGTGCAGCGCCCCTGGG
GGGCCCCCTGCTGCCTCCGACCTACCAGCATCACCCCCCTGTTTGTCTGCCCCCGGGCCCGCTGTTGCAGCCCCCCTA
CGCCGCCCTCTATGCCGCCGCTCTGCATGGATTGTTGGCTCCACCCCTTCACCACTTTTGCAGCCTCTCGATTTGGAG
GCCATGGCCAGGCAGCGGTTTCAGCATTTATCTGG¶

>*NlFoxC*¶

TCCACTGCTCCTCGTAGACGTACAATTTTTTAGTGAATGTTTATCGAGTCTCCTCCTCATCTTTTATCTTGGGAACGATG
CTGCACTGATGCTGCACTGCTCCCCCCGGTCTCTGTAATCCGGCACCCGGGAACATCCGGGCGGCGGGCACCATGCA
TGCCCTGTTCGGCGACCAGAACGCCTATTACGGCCGACAGACCGGCTACGGACCCGTACCGGCCGGGCCCGGACAC
AATCCGTACACCACCTACGAAAACTACGCGCGATACGGATATCCGGGTCCCTATGCTCACCAACAGTCTCTCCACCA
TGGCCCGTCCAAAGACATGGTTAAGCCTCCTTACTCGTACATAGCTCTTATCGCAATGGCTATACAGAATGCACCTGA
TAAACGGTGTACGCTAAACGGGATCTATCAGTTCATAATGGAACGGTTTCCGTACTACCGCGAGAACAAGCAGGGAT
GGCAGAACTCCATCAGGCATAATTTGAGTCTCAACGAGTGTTTTGTCAAGGTGCTGAGAGACGACAAAAAACCAGGC
AAAGGCAGCTACTGGACACTGGACCCTGACTCCTACAACATGTTCGACAACGGCTCCTACCTGCGTCGCCGGCGACG
CTTCAAGAAGAAGGACGCC¶

>*NlFoxD*¶

GTAATGTTGTTGACAGCGTCAGTGGCTCCCATGTGCGTCGCCATGCAAGACCGACATCACCGCTACGAGGTGACGCC
GCTCGATCTGCGACCCGCCGTCACGACGCCACCGCACTCCGACGACATGGACGAAATGGACTCCGACGAGGATATC
AACGTGGACTCGGATGGTGAGCCCGACTCGCGACTCCTCGGCTCGGATGCCCTCGACTTCCATCGCCGCTACATGCA
GCAGCAAGCACAGCCGGGTGACGACTCTCCCGGGTCGCCGTCACGCGGAGGCGATGACTCCTGCTCCACTGACAAG
AAGTCTTCGTCGTCGTCTCTAGTGAAGCCGCCCTACTCGTACATTGCTCTGATTACGATGGCCATCCTGCAGTCGCCGC
ACAAGAAGCTGACGCTCAGCGGTATTTGTGAGTTTATAATGACACGTTTTCCGTACTACCGTGAAAAGTTCCCCGCCT
GGCAGAATTCTATCAGACATAACCTGTCACTCAACGACATAACCTGTCACTCAACGATTGTTTCATTAAGATTCCACG
TGAACCGGGAAATCCAGGGAAAGGTAATTACTGGACCCCGGACCCTATGGCGGAGGATATGTTTGACAACGGCAGT
TTCCTGCGTAGGAGGAAACGGTACAAGCGACAACCGCCCGACTTTCTGTTGCGGGACCCGCACGCAGCTATGGCTTT
CCTGGCACCACCCGACCGCTTCAACCCGCTGCATCCTCACACGCTTCACTACCCATATCTGTCGCCGCTGCCTCCAGC
CGTGCCTCTCCTGCCTCCCGCTGAACTCGCCCGCCTCTCGCTCGGACTCAACCTGCTCTCGTCGTCAGGGCCGCCCCCG
ACGTCTCTCCCTTGCAAGCCGGTCCCAGTGCAGGCGGCACCTCGCTCAGCAGCCGGCTTCAGCATCGACTCCCTCATC
GGCAAGAACCCTCGCCCGAACAAGCCTTCGCTTCCGCCGCCTCCACCGCCTCCATCCTCGCAGCACTTCCACCACCTG
CCGACCTCATCGCCGCCGCCCGTCTCCCGGAGCATCGAGTCGGCTTTCACGCCCCTGGCGGCCGCCGCCTGGGCCAG
GTGAGTGACGGGGCTCACGCTGCCACCACCTTATCACTCCTAG¶

>*NlFoxF*

TTCTCGTGTTGTTGATACTCGCGCGTTGATAAATAAAAACGAAAATGGTAAAAATACAGGAACTGTACAACAGTCCA
GTACCGACACGGACCCCTCGCAGTACCATGGAGTGCTCCATCAAAGCATCAACATCCAGCAACACCTCACTCATCT
ACACCTTCACGCGTCTTTACCTTCCCTGCAGGGATCCACAGGATCCAACCCAGGCCTACCATCCACCAACTACGAGC
GACCTCAGCCAGACTCGATCATTGTCGAACCCACGTCAGCATCCTCTCAGCTCACCAAGAATGGCTCTCCCCGGAAA
CCAGCAACCCGAAGACCCGAAAAGCCGCGATACTCGTACATTGCTCTCATCACAATGGCCATTCAAAGCTCCGACAC
CAAACAGATGAGACTCAGTGAGATCTATGATTACCTGCAGCAGCGATTCGAGTTCTTCCGCGGAGCCTACACTGGCT
GGAAGAATTCCGTGCGACATAACCTCAGTCTCAACGACTGTTTTATCAAGGTGGATAAGTCGGTATCACGGCCGTCG
AAAGGCCACTACTGGAGACTGCGCCACGACTGCAACATCCTGGAAAACGGCTCGTTCCAGCGCAGACGTCGCGGCA
CCGGTCGCAAAAAGCCCCCTGCGGGCCCCCCGGGGGGCGTCCAGAAGGGGGTGCTGCCAATGCAGCTCTTCCCCTGC
AGCGTGGACCCCGCTGATCGCATCAGCTCCTCGCCCTCCACCGCCATCTTGGACTATCAACACCCGCACCCGCATTCA
CATCACCATCACCATCATCATCAGCAGCAGCAGGCGCTCTATCAGCACCTACCGACCGCCAGTGGCGGCTATCAAGT
ATTTCAGAGCCCTTACGAGCAACTGCACCAATACTCTACTGCAGGCTACCCAGATCCCACAGCACAGCTGTCTTTGCA
ATGGTCCGCCGCCGAAAACAATTTCGACTGCCTGGCAGCCTACAATAACGCGGCCTACAACAATTCCAACAGCACCT
CACCACCTGACACCATGGACAATGGTTTCAGGGAGAGAAAGCCGTTTGCACCCCTCACGCCTTCTCCCCCTCCCTTAA
CGCCCGCTTTTGCAGTTGCTGCCACTTATGGCAGAGGTCAAATGAATCCAAGCATTCAACAGTACGAGATGATCAAG
GATTACAAGGATTACCATTGATCAAGCAGGAAAAACTGTCTTCAAATGTGAAACTTTTTAGAGTAAGATTCAAGTAA
CGTTCAAATAGAAACTATTAAGATATAAGAATAAAACTAAAATTTAGGAACATTTTTATATGAAAGTGCAGGATTCA
ATGCTGTTCAAC

>*NlFoxG*

TCAGTTCGAGAGGATATTCGAATTAAATTCTCTCCAAGTGCAGTTTTCTCTCGTGTTGTGTACGAAGTTCCCATAGAGT
CACAGTGGAGGAAAACAGTATGGTGAAGATAGACATTATCGAAAACCCCATAATGGCCCGTACGCCTTTAAAATCA
TCGTTCAGTATCAGTTCCATTCTCCCAGAGACGGCCAGTCGACCACCCAGTCCGCTGTCATCACCTGGCATCAGTTCC
TCGGCGGAGATGAGCGACGTAGACGTGGAATCCGATCTGGATGTAACGGGGGATGGAGATACACCGCCACCCCTCG
ACTGCACCCTGTCCTCCTCACCGAAAGGAGAAGGTAAAGACGGGAAGACGACTGTCGAAAATGGGAAGACAAATG
AGGAGAAGAAGAAAAACGAGAAGCCACCCTACAGTTACAACGCGTTAATCATGATGGCGATCAGACAGAGCCCCG
AGAAGAGGTTAACTTTGAACGGAATCTACGAATACATCATGAGGAATTTCCCGTACTACCGTGACAACAAACAAGGC
TGGCAGAACTCCATCAGACATAACCTCAGTTTGAACAAATGTTTTGTGAAAGTACCTCGCCATTACGACGACCCCGGT
AAAGGCAACTACTGGATGTTGGACCCCAGCAGTGAGGATGTGTTCATCGGAGGTACCACGGGGAAGTTGAGGAGAA
GGTCGACCGCAGCCTCCAGGAGTCGCCTGGCAGCCTTCAAGAGGACGGTCGTCCTGGGAGCGGCAGGTCTCTACCCG
GGGGCACCAGGAGGCCCCTTCACGAATCCCGCGGGATGGGGGATATCACCCTTGGGACAACTCTACTGCAACCCGG
CTGCCTTGTACCATCGTTACCCGGGTTACCCATACCCTGGACTTCTACAACCTCTACCAAAACCGACACCCATCCCCG
CACATGCGTTCTCCATGGAGCGACTCCTGGGTGACACCACGACGAGCACCCCGACCCCTAGTGGACCGCCTCCAGCT
TCTATCGCCCTCAGACCCTCCGTGTATCCTCCCGGGTACCAGGACATCTACGCCACCCTCAGGCATCACCAGCAGATG
GTGGCGGCGAGTTTACAACACCAGACGCAACCTCCAGTTACGACCTCGGCCTCCTCTAGTCCTGAGCCTCCACGGAG
CCTCTTCAAGCCTGTCACCGTCCTCACACCCCGGCCCTCCAGCTAGGATACTCGTCATCTGTAAGTCGTTGCTA

>NlFoxJ1¶

TAGCGACGAAGACCAATGCAGCAATGACCATCAAATGATTCTGCAGCCTACAAACGATGAAGACCGATGCGGGAAC
GGCCATCAACTGATTCTGCAGCCTATAAAAGACGAAGACCGATGCGGCAACGACCATAAACTGATTCCGCAGCCAG
CTAGCGACGAAGACCGATGCGATCTCACCAGTCTCCACTGGTTGCACCAATCAGACCTCCTCAACAGCTGTGTCTCCT
CACTCCCCACGCCTCCCAATTCTCCCTCCCACGCGCACACACCCTCTCACTCACTCGCCAAGGCCGCCAACGGACATA
AGGCCGCACAGACTCTAAGGCTGACACTCAATCCAGCCTGTGCCGAAGACTACAAACTGAGCGGGGATCACAAGCC
GCCATTTTCGTATGCCACCCTCATCTGTATGGCTATGAGGGCCAACAATAATAAGATGACCCTATCTGCCATCTATGC
ATGGATCAGGGATAACTTCCTTTACTACAGGAACGCTGATCCTACATGGCAGAACTCGATAAGACACAACCTATCTC
TGAACAAGTGCTTTGTAAAAGTGCCTCGGTCAAGGGACGAGCCTGGTAAGGGAGGATTCTGGAAGCTGGACTTGGAT
CGTCTGGAGGAAGAGCTCATGGTTATGACGTCACCGTCAGATCCTCCCCTCACGCCGCCAAACCAGCCACCAGTCAG
CGAAAGCCCCTCTACAATCATGCCGCCTCCCTCGCTAACCCTCAACCTTACCAACTTGAACATGACTGTGGAAAGCAT
TCTGCCAACATTCCAAACTGAGACGTGTTTCGCTGAAGATGAGTTGGCTAACCTGCTTGTGGGTAATGGCGGGTGGGA
TGATTCTCAACTCGAGCTCTTGCATTCTCTTCTAGATACTATTTAGATGTTTTAGTTATTTTCCATTTTCGAACAAGAAT
TTGACTCTCAACTATGGTTGAGTTAAAAGTTCTTCCGACTTGAAGGGATATGCGGAGCATAGAACAGTAGGGATACA¶

>NlFoxJ2¶

CTCTAGTTGAAAGTAGGGTATCACCAGTTTTTGGAAGTTATGTGGATTTATGATGACGAGAAAATAGTTAGAACTAAA
TTCACAATCAGGGTGTGGCATGCTAGCAGCATCAGGTGATGGCCGATCTAGCCGGCGTTCGCACCGCGACCTGTGAT
TGGCTCAACTCTCCCCACCCTTCCTGCACACCCTCTCCGCCATCCCGGCATCCTCTTGCTCCTCCTCCCACTCTCAACTC
TCCCTCCCACCTCCACCATCACCAGCTCTCCTCCTCCACACCCTCTCCACCCTCTTCCTCCTCGTCGGCCTCGCAGCGA
CAGTCGCGCGATGGAAAGCCGCCCTACAGTTATGCAAGTTTGATACGCCTGGCCATTAGTAACTCAGTGCAGGGAAA
AATGACGCTATCCGAAATTTACCAATTCATCATACAAACGTTCCCTTACTACAGAGACGCCAGCACCGGCTGGAAGA
ACTCGATCCGGCACAACCTGTCTCTGAACAAGTGCTTCACAAAAGTGGCCCGACCCAAGGACGACCCGGGTAAGGG
TAGCTACTGGGCCATAGACCACACTCACAGCCAAGATGGCGGCGGACAACAAGATGGCGGCGTCCCACGCAAAAAG
GTCAAGCTGCCCAGGGTATCTCCCTACAGCCCAGAATGTAACAGTAACAGCAGTGATGGTAGATTAACATTGAAGAT
GGCCAGAAGCAGTACAACTCCAACCCCTAGTGAAAACTGTCACAGTTTGGATGAGTTCAGCCTGGGAGACAGCAAA
GAGGTGTCAGCTGTCATTTCCAAACTACTCTCCCACTATGGTGTTGACCCTAGTGACAGTCAGAGTGACTACCAGCCT
TGCCAAACCGACTTCCAGGAGAAGAGTGACTACCAGGGCTGTCAGACTGACTACCAGGCTAACAGAACTGGTGCAT
CATCGTCTTCATCCGACTACTTTCATCCCACACCTGACACCAATTTGGAACCTTCCCAAAACTACTACCAGGAGTTTCC
ACAGTTGGTCGACAACAACAGTGTTGCCAACACTCTGCCAACTTACAGTGAAAACTCAAGGTATGCTGCAGATGTGC
CCAATATGGCAGATGAAAGTCTGAGGTATGGAGTGGGAGAGAGTGCTTCAAGGTATGGAACTAGTAGTAATAGGAG
TATAGAGGAAAATAGTTTCAAGTACAACAATTTCAGCCCATACAAAGCCTCGAATAATTTTGAATCAGGTAGTTCTA
GTAGTTGTTATTACAATAATCATCAACAAAACCAAAACTCCGATTACTATTACAGTGGGTTGAAATCAACTTCCTATG
AGTATATAAGGGTGAATGAGGAGGATAGATGTGGCAGTGCAGGGGGACATTATGTGGGAAACAATAACAAACCAGT
GTTGCCAGAGGGAAATGTGGCAACGTTTGAGACTGGCGCCCAACGTGGGACCACAGCGCCTTCCTATTGGTCGTCGC
CAGCCACCGACCAATCATGTCGCTCGCCCTACTGTGCATCCGGCTCCCCATTGGCCCACGGCTCGGCCAATGGCGAG
GCGTCTCTGTCCCCCCACTACAACCAGATGGCGCCTGATTTGAATGGTGCAATGATGGCGTCTTCCTTTGATTTCGGCG
CCAATTCAAAATGGGACACTTTGTTATAGCAACGTTTGTTTGTTTAGTAAACAAAACTGCCGTGTAAAGTAAATCAAT
TTGTAGGTTAGTATAATTAATGTGAGAGATTGATTGATTCATACATTGCAAGGGAGAGTAAAATGGGTGAATAATCTT
GATATTCATGCAAATAGATTTTTGTCTTGTTCTAACTACCTCG¶

>*NlFoxK*

GACTGTGAAGACGTAATGTCCGCACATTCACGGTCTCAAGAGAGCGATGCATGGGCTCTTTTAGCCCTAAAATCAGC
TCCTTCGAGTCCAGCAAAGCTACAATGGAACCCAGATAATAAAACTACGCCGATTGCAAGATTAGAAGGTCGTGAAT
TTGAGTATATGGTTAGGCAGAAACGAATCATTATAGGACGAAATAGCTCACGAGGCGAGGTCGATGTAAACATGGG
ACATTCAAGTTTTATTTCTCGAAAACATCTTGAATTGTTTTTTGAACATCCTAATTTCTTCATGGTTTGTAATGGAAAAA
ATGGAGTTTTCATCGATGGTGTGTTTCAACGAAAAGGAGCTCCAGCATTGCAACTAGCAAAATCATGCACGTTCAGAT
TTCCGAGTATGCCGATCCGCCTAGTGTTCCAGTCTCTGGTGGACGAAGCTGACCCCCCGCTGCACGTCCCGCCTCCCG
CCAAGCAGCGACCCCCACTACCTCCGTTGCGGATCAACATACCTGAGCCAGGTGCCAACTTCACCAGTCCCTTCCCAT
CTCCCACTGGCACTATCAGTGCGGCGAACTCGTGCCCGGCGAGTCCGCGGTCGCACGGGCCGCGTGGCAGTGGTCGC
AACATCACGTCCGACCTGCAGATGGTGGCCGCATATGCGGCTGCAGTCGCCGGGCCCAACTCGGTCATCAACACTGT
CACCGTTTCCACGTCCAACAATCTCGACGATCGAAACGAAATGCTTATAGATGGATCAAGCCAGCAGAACGACGAC
ATTCATCAGAATGATGGCAAGCTGTTCCGTCCTGCGAACGGCTGCTCGTCTGATTACAACACCCCACCCAAAGAAGA
GACAAAGCCGCCGTACTCTTATGCTCAGCTAATCGTTCAAGCTGTGGCCTCTGCCCCCGATAAGCAGCTTACTCTTAG
TGGTATTTACTCTTACATCACCAAAAACTACCCCTATTATAGAACAGCCGACAAAGGTTGGCAGAACTCTATCCGCCA
CAACCTGTCGCTGAACCGGTACTTTGTGAAGGTGCCGCGCAGCCAGGAGGAGCCCGGCAAGGGTTCGTTCTGGCGCA
TCGAGCCGCAGTCCGAAGAGAAGCTCATTGAGCAGTCGTTTCGGAGGCGGCGACAGCGCGGTGTTCCTTGCTTTAGA
GTTCCTTTCGGCCTGTCATCGAGGTCGGCGCCTGCTTCTCCAAGCCACGTGGGCATGAGTGGCTTAATGACGCCGGAA
TCACTGTCCCGAGAAGGCTCTCCCACTCCCGACAGCACAGTCATCTCACCGGTCGTCACAAGTCAGTCGGCGCCCGG
ATCTCCAGGACATCCTGGTGTTACATTTGTGAGCACCAGTAATAACATCTCAGTGCCAAGCGGCAACATCGCAATGA
TTAACAAGTCACGCATTGTTCAGCATGTCACTTTAGTCCCCAATGGCGACACTAGTAGAGAAGAAAAATACCTAACA
ACAGGCCACATGTCTGGAAGTAGCATGCTTAGTATAGCAGAAGAGCGTTCAATGTCTCCTCCAGTTATTGTTCAAGCC
GCATACACAGGGGCATTTGTTGGTGAGGGAGGCATGAAGCGGACCTTGGACGATGGCGATGCTAACGAGAGTTGCG
GACCACCCGTCAAACAGCTACGAGAGAGCGATTCCGATACTGATCATTCATAATCTGTCATGACAAAGAAGAGTGC
ATTCGTAGTTGACAACTCCTGCAATGGCGGCATGCCGATGGCCGAGTGTGTGTGGCGCATGCCACCCAGTGACCAGT
GTAAACCGGACCTAGTTTGTGTCTAGTGGCAGTG

>NlFoxL1¶

CAATCTTCGTTCAAGTGGAAAGGAGTCACGCGGGGAGATACCATAGAGTTTTCATCGTAGGATTGGAAAATTATAAC
TGTCCGATTGAGTGCGTCAGAAGTAAAACGTTTTGGGAAAGGTAGTAATTTCTTGAATCGGTGTTGGTTGAGGAGTTG
ATTAGGACAGCTGATGAATCGATGATGCTTCCAACCTCAACTGCCAGCGCATCGCAATCAGGTTTGGATCTGTCCATC
ACAGCCGGCTCTCAACCGTTTCAGCAGTCGGATCGCAGCCAATCTTCGAGTCCCGTCGAGGGTCAACATCAGTTGCTG
AGTCAGCAACAGATCTCGCCCAGACTAGAGCAGCATCCAGGAGCCGCAGACCTCTCGACATCTGAGTCAGCGATAT
CGTCTTCGGCAGCCATGGACCTCCATCACCATTCGCCTCCACACCTGGCGACCGCCTTCCCCTTCCCTGCCACTCATCT
GCCTTATCTCTACCACCATTACAACCTGCAGGCTCGGCTCCTGCCCTACATACATCAGCTGCAGATGTCTCACTTCAA
ACAGACAGCAGCTACAGCGAGCAGAGAGCGTCCCGAGAAGCCACCCTACTCATACATAGCGCTGATAGCAATGGCG
ATCACTTCGGCGCCCAATCAGCGACTGACCCTGAGCGGCATCTACCACTACATCATCGAGCACTTTCCCTACTACCGC
GACAACTGCCAGGGCTGGCAGAACTCGATCCGCCACAACCTCAGCCTCAACCCGTGCTTCGTGCGGGTGCCTCGCGA
AAAGGGCGAGGGCAACGGCAAGGGTAGCTACTGGACGGTTGATCCGGTCGCTGCCGCCAACATGTTTGAGCGAGGC
AACTATCGCCGGCGCAAGGCGCAGCGGCAAGCCCCGGCGTCACGGGCTACTGTGCAACAGGTTACATCAAGTCCAG
AGGAGAGCGTCCGAGTCAAAGTAATTGAGAAGAACGACTTGAAAAACAACGATAGTAATACGATAACAATTGAAAT
TGAAACAAATGAAGATGCAGCACCGTTGAAGAAGCCAAGACTCGCCAATGAATTTGCGTCGGAAAACACGACTGCT
CCCTTCACACCAGCATCGCGACTAGGCAAAACATCGATTTTTCTAATCGAGAATCTCATGAAACCTGAGCCCAATAG
CAATTGCTCATCAGATGAGCATAGCATTGATTCTGTGAGGAATTCTACATGAAGATATAAATAGGCTGTTGTGAGATC
A¶

>NlFoxL2¶

GTATTGTGGAGTGGTTGGAGTGAGCTGTAACTGTGGTTTCGAACATTGTGTTAAAGTGAAGCGTCAAATTTCAGTCAT
CATAAGACGTCATGGTGTCTCATCTGCTCCCAGACTCGCAGGATCTTGAAGACAATGAGCCTCTCAAAATCAAAGAA
GAACCGTTCATCACCCGTACCGCGGATTGCTCCAGCTCCCACATTCATGGCTACGACGACAAGAGCAGCGACAACGT
GAAGAGTTCTCCGTGCTCCTCCTCGTCACCGAGCAGCCACACCCATCAGCTGCAGCACAGAAGCAGCAGCAGCTGCA
GTGGAGGCAGCTCCGGCGACCATCACCTGCTCGCTCAGCTGCAGTCCACTGTCTCCTCCACTGATCTCATGCCCTACA
TCCACAGCCCCAGCCCCATCCAACACACCACCAGCAAGGAGTCCACTGTTGTTTCCACATCAAGCGGCTCCCCCTCG
CCGGGAACAGTGGACTCGGACCCCTCGGTGAAGCCCCCCTACTCGTACGTGGCTATGATCTTCTTCGCGATCAACCAA
TCGACCCTGAAGCGAGCCACCCTCGCAGAGATCTACAACTACATCTCGACCCGCTTTCCCTACTACGACAAGAACAA
GAAGGGCTGGCAGAACTCCATCCGCCACAACCTGTCGCTCAACAAGTGTTTCATCAGGGTGCAGCGGGAGGGCGGA
GATCGCAAGTGCAGCTACTGGACGCTACACCCGAGCAGCGGCGATATGTTTGAGAACGGCAACTTCAAGCGTCGAC
GCCGTATGAAAAAGACGCCGTTCGATGTCGGAGGCGGCTCTCCCTACATGAAGCAACTATACGCCGACTCGCCATAC
CACCCCCACAACCACCATGCCGCCCCCGCTCACCCACACCCCCACCACCTGTTCGCCACCAGCAATCATCATCATGC
CGCTGCTGCTGCTGCTGCCGCCGCCGCTTACGGCAGCCATTACACCAGCAGTACATGGCCAATGCAGCCTTCACAGTT
GACCTACCCATCTTGTCAGGGTATTGTTACTCGCAACGCTTCTCAGTATCCTATCCAATCTCAGTTGTCTGGACAGTTG
ATTCAACCTTTCCAAATATCAAGTCAATTGAACAACAACTACCAACTGAATCCTGGAATAAGTATGACCAGCAGTAG
TGAGTTCTCATCCTGCTCCATGAGAAGAACAGAGTCTGATTCTATGCGATATCCGTACTGGACATCTTCAGAAATGAA
AGATGAATCGCCGATCTCAATGAACAGCTCGCCAAGTGAAAGCCCGTACAACGCTCTAAGTATGGAGCTGTTAGCAT
CCGCCAGACCAAAGTGTTTCATGTCGTGAATGATGCTGGTATTTGTTACCGCGGCTCTAGAGCGGTTTCCACTCTTTGG
AATCAAGCGATCATCTCAGTC¶

>NlFoxN1

GGACGGTATCGTGTATAAATAGTGTAGTTTTACATTTATTTTAGAGTTATTTATGTGAAATAAGTGAATATATGAATTG
TGAGTGTAGTGTTAAGTAACTGTGAAGATGGATATGTACTTAAGCCCTCCTGACAGTTTCAATCTGCAGGAGATGCTC
GATTGTGATATCAAATGTGAGATGGCAGATGGGTTCGGCCTCGACTGCGACCTACCGCCCCTCGACCTAGAGGATGA
ATCACACCACGCGTGGATCCATGGCAACTCGAACTCTAGTTTCGAGTTGGACTTTTTTGGCACCGACTCGAGCGCCTC
ACTCATGGTCAATCCGCACGCCATCATGCCCCTACCCTCCCTGCAGCACCTCAGCACAATTCGATCAGCTGATGTCGG
CAGTCGATCAGCTGATCTCGGCGGCTGTGACGTCATCAAGGTCGAACTCCCCTCCACGCCGCCATCACCCGATGATCG
GTCTGACCATTTGATGGGAGACGACGAACCAGCAGAAGAGGAAGAAGAAGAAGACAACGAAGAGGAAGAACCGG
AAGACGATGATGATGATGAGGACTACCAGGAAGAGAGGAAGCCAAAGTCGTTGAGAGTGGATCCCCTGAAAGTGTC
ACTAAGTGCCAGCTCAGTGGCAAGCAAGCAGCTGGCCAGTATCACTGCCAACGCCAACACACCCCACGGCTCAATC
AAGAGTGAGACTGCTGCCAGTCCTTACACACCCCCCACCACTACCAAGAGTAAGAGGCTCATCGTGAGTTCGAGAAA
ACAGCTGTGTCTAAATGATGCCAATCTCTATCCGAAGCCAGCCTACTCGTATTCGTGTCTTATAGCCTTGGCTTTGAAA
AATCCACCGTCTGGAAGTTTACCCGTTTCAGAAATCTACAATTTTATGTGCAAGCACTTTCCGTACTTCAAGTCGGCGC
CGAACGGCTGGAAGAATTCGGTGCGCCACAACCTGTCGCTCAACAAGTGCTTCGAGAAGAACGAGAAGCCGATGGC
GGGCGGTGAGGGGGCCGGAGGGGGGTCACAGCGAAAAGGCTGTCTGTGGGCGATGAACCCGCACCGGCTGGCCAA
GATGGACGACGCAGTGGCCAAGTGGTCGCGCAAGGATCCGTTGGCGATACGCCGGTCTATGCTCAATCCAGATGACC
TGGAGTCGCTGGAACGAGGAGATTTGCACGTGGTCTGGATACCCGGTGACGGGTGTGGGGAGGGAGATGACTCGAC
AGGGGAGGACCAAGATGGCGGCGCTCGTGACCCCCTAATGGCCGCCACTGCTCCCCCCGCCACCGCCCCCGCAGTGC
AGCCGCAGCCCAAAGTGGCGCCAATCAGGATTAGGACGGTAGCGGCCAAAGTTAGACCGCAACCTACCGCAACTAG
CACAGTAACCGCCACAACCGCAGCTGAGGTTCTAAGACAAGAAGTGGTTCACCAGCCATCACACGTAGTGAAACAC
GAAGTGGTGGAACACCACGATTACGAAGAGGAAGAGGAGGAAGCGGAGGAGGAGGAGGATGAAGATGAGGATGA
GGACGAAGAGGAGGAGGAAGAGGAAGAAGAAGAGGACGAGGAAGAGGAGGAGGAGGAGGATGATGGGCCTCAG
CCGATTGGGAGTGATGAGAGTGAGGATGAGTTGAGCATGATGCCCCCCTCCCTGCAGCCTTTCGAGCCCAGGTTGGC
GCTGGCCAAGAAGTTCACCGAAGCTCAGAAGGAGTTGAGGTTAGGTAGCGGTGGGCAGCAGCATGATATCGAGTTG
ACAAACGACATCCTTGAATCACTGAACTGGGTGGATCTTCCGCGCTACGAGTCGCCCGGCTCCAGGTCGCTGGACGT
GACCGTGCCTGTCTCCGACTACTCGGACGTGTACGCGGCGAATGTGCGAACGATCGCGAACGTGCGAACAATCGCCA
CCGCCCCCGGCGTGGTCGCCGCCTCCCGCAAGCGTCCCGCCCCCACACGCATACAGGGCAACTACGTCTGTGTTAAG
CGCAAACTGCCTCAGGTTAACTGAACTCGTCCTACTTTTAAGATTTAAGAGGGTCTAGCGTTTTACCGATGCAACAGT
TGATCGGTATGTGAAGTGAACGAATACTAAGTTTTATGTCATTCTAATCTACTTTTTGTTTTAAGTTGTAAGAGGGTCT
AGCGTTTTACCGATGCAACAATTGGTCGGTATGTGAAGTG

>NlFoxN2¶

TGTGATTGCTGCTAATGTGTGTTCAATACCGGATAGGATTAACTTAAAACAAGTGACATGACACAAAATGATTGCAT
GGACTCCAAACGTGAGGACGTCAAGCTGAAGGAGATGAGGATGGCCCCTGACCGTGTATGTGACGACGAGGGCCCC
TGTGCCCTAGGGGGAGGCGCGCCCCCAGGGGTGGTGGCCGGGGGTGCCATCAGCCCCCCTCACCCGGTGGCGCTCAT
CACTTCGGCCGCCCTGCACCTGCAGATGAAGGAGGGTGAGGAGGGCGGGCCTCCCCTCTCCTCACCATCACCCACCG
AGCAAAAGGGGGGAACCGAAGATGATGACCTAACCTCGTTGAGCTGGTTGCTACAGGATAAGAATATTTTGAAAGG
TATAAATCTGCGAACAGCTCCGTCCGGAGTGCAGGAATCACCGACTAGCGAGGACAGTGCTAGTGAACAGGGGGAG
GTGGCCGAGGGGGAAGGAGATTCCACATGTTCCAGTGTCAACTCGTCGAGTCCTGTGCCTCCCAGTGGAGGGGCTGG
TGGAGGTGGAGGAGGAGGAGGAGGGGATAGAGAGAGAGTATTAGGAGTGACGAACTGTGTCATGAAGAACAAGCA
TCCTCATCACATACCCTACGATCCCAAAGTCCATATAACGAGCAAGCCGCCCTACTCGTTCTCGTGCCTCATCTTCAT
GGCGATCGAGGACTCACCCGTGAAGGCGCTGCCTGTGAAGGAGATCTACGCCTGGATCCTCGATCACTTCCCCTACT
ACAAGAACGCGCCCACCGGCTGGAAGAACTCAGTGCGTCACAACCTGTCGCTCAACAAGTGCTTCTGCAAAGTCGA
AAAGGCACCTAACCTAGGCAAGGGCTCTCTATGGATGGTGGACCCAGTGTACAGGCCTAACCTGGTGCAAGCCCTTA
AGAAAGCACCCTACCACCCGTATGCTGTCACGGGTTCTGGCTCGGATCGGTCCACACCCACCAGCAACTCTCAGCAA
CCTGACAGTTTATCGTCAGGCGAGCACACACCGGACGACACGCCTGCACAGTCGAAGGCTGGCAGTAACGGTCGGC
TACTGCCCGACCCTGACCTGTTCCCCTACCTGAGCCGGCGGCTGCTAGCAGCAGCTGCGGTGGCCGCGGTCTCCGCCG
ACTGCCGCTTTGACGCGGACGCGGAAGCGGAAGCGGAAGACGACGGATCCAACTCGAGTCCGCTGGACGATGTGGA
CGCGGCCGCCGCCATGTTGGCACTCAAACACGGCCCGCATGTGCGCATGAGCATGGAGCAGCGGAAAGACGAGTAC
CACAACCAGTTGACGGCTGTCAATCTAACTTCCAAAACAAAAAAGAAGAGCAAAGAAGAGGAGGAGAAAGACGTG
CTGATAACATCGTGTCCGAGTGCTGACCACACCTACAGCGTGGAGCACACACAGGCGCTTCACACCGACATGTACAC
GCACAGGTTCCACAGCCCATCCAAGAAAGATCTAATCTCGCCTGAGAAATCCTTCACTGACAGCCAAGACTCCAATT
CAAGTTGGGCGATGCGAGCAGTTCGCGCGGCGAACGGCCATCGCAGTTCACTGGGCGATCAGGAGGAACTGCGCAA
GATAGCCGAGGGCGCCGACGCACTTTTGAATTTGGCGGGCTTTACCACCAGTCTGCGCAGTCGATCACGCAGCTCCA
CTGCGCACCGAGCCAAACGACAACGTCTAGAGGAGGAGGAGGAGGAGGCGTTGGTGGACGAGGAGGAGGAGGGTC
GGATGCAGGTGGAGGAGGTGCAGGAGGTGGTGGAGCGAGTTCCGTTCAGGCCACGGCTGTTGAGGAAAAGTCCGCT
GAAACGCGACTTGAAGAGCGAGAGACGCGAGGAACGAAAACAGCGCGAGAAAAAGGCCTGCATGAAAGTGTTGGA
CTACGCGCAGGCCATCATGGATTCAATGAACGAGACCAACAACAACAACCAGCAGACTGCGTCAAAGTCGGCCGAC
GACAAAGTGGTCGCTGACAAAGTGGTACCACCACCAACCGATGACAAAGTGGTGTCGTCTTCTTCCACATCTCACCG
CGGCGACAAAGCGAGACGAAAGTCGCTGTTGACCCCCGTCAAGCACTTGGCCGCCAAAGTGAAAAGGTAGGGACC
GGTTTCCGGGGGTCGAGTTGTCGGCCTCTATCTTGCGCGCTTCTCGTAGTAAAATTAACGTAAATAACTAGTGAAGTA
GTGTGTTTTTGTGTACATTGTTGCAGTGGTAGGTT¶

>*NlFoxO*

AGAGATGTCCGAGTGAGAGGATTGATAAGTTTGCAGACTTGGAAGTAATCTTGTGATAGGTTTTTGGTGTTGAAATGA
TGGAACCGTTGACCGACCTGGGCTTCGAGCCCCAACAGCGTGCCAGATCAAACACTTGGCCCCTTCCACGACCCGAT
GACGTACCCGGGGCAGAGACAGGGGGCGCCCCGGGACTGATGGGGGCCGTTGTGCCCCCTGCAGCTGCTGCAGCCC
AACTCAAGAAGAACTCGAGTCGTCGCAACGCGTGGGGCAATCTGTCCTACGCTGACCTCATCACTCAGGCGATCGCG
TCCGCGCCCGACAAACGACTCACCTTGTCGCAGATCTACGAGTGGATGGTGCAGAATGTGCCCTATTTCAAGGACAA
GGGCGACAGCAATAGCTCTGCTGGATGGAAGAACTCGATCAGACACAACCTATCGCTGCACAACCGGTTCATGCGC
GTACAGAACGAGGGCACGGGGAAGTCGTCGTGGTGGATGATCAACCCGGACGCCAAGCCGGGCAAATCAGCTCGCC
GTCGAGCCACCTCCATGGAGACGTCCAAGTTCGAGAAGCGGCGAGGCCGAGTCAAGAAGAAGGTGGAGGCGCTGA
GGAACGGACTCGACGCGACTCCTAGTCCCAGTTCATCCATTTCCGAGAGCCTTGATCTGTTCCCTGAATCGCCGCTGC
CTGGCTTCCAACTAAGTCCCGACTTCCGACCCCGAGCGTCGTCGAACGCATCGAGCTGCGGACGCCTGTCGCCGATTC
CGGCCGTCGAGACGGACTGGACGTCCTACAGCTACAACCCGGAACAACTGGCCGGCAGTCTCGAACAAACGATGCG
CCTGGGGGTTCCCGCCACCGAGGCCTCCGTTGCGCCACCCCAGTTCATGTACCCCACACCGCCCCCCTATCACCAGCC
CCCCTACCATTCCTACACCCCCCAGCAGCCCCCGCCCCACCACTGTCCAATACACAACACCAGTCCACAGCAGCCCT
GCGCCTGTAACCACCAAACCACTCCCAAACAGAGCATCTCACCGTCGTACCCGCAGTCACCGAGCCCCAGTCCGCCG
GCAACAACGGTGATGGGTCAGCTGATGGGCGCCCTCAACCCGTCCCTTATCGACGACCTCAACCTCAACATAGAGAC
GTTCGACGGATTCGACTGCAACGTCGACGAGGTCATCAAGCACGAGCTCAGCATGGAGGGCAGCCTCGACTTCAACT
TCACGCAGCAGCTGCAGCTGCAACAACAGCAACAACAGCTGCAACTGCAACAACAGCAGGATCCGGCCACCGCTAG
CCAGCCGCCTGTCTACTCCGGCCAGTCCTGGGTTCATTAGCGGTACGTCAATGGTAATCGAACACTACGTGGAATCA
ATTGCAAGGATTTCAATTATTACTCCGGTTATCCAAATCGTTTTGGAAGACCATAATGCTCAGA

>*NIFOXP*

GCAGCATAGTATTGGTCTGCAGCCAGCTGCGGCCGTTTTACAGCAGCATGCCAAGATGCAGCATATAGCTGGAGGGT
TAGCCCAGGCGGAGATGGTATCGTTATGGAATAACTTTCAAGTTGATGGAACGGACCTGGACCAGAAGCAAATCGAC
AAAACAACGGCTGGTTTAAACGGAATACTAGCTTCAATGGGTATAGGAAGAAGAGGAGCTGGTGGAGGAGGAGACT
GTTTAAATGGTGGCAGTGGCAGTACACCTCAGGAGGACACCAGCAGTTGTGCAGATGCCAACCCAAGCTCCACCAAT
AAAGTCCACCTACTCTTTGGTCACGGAGTATGCAAATGGCCCGGCTGTGAAACCGTCTGTGATGATATACAAGCATTT
TTCAAGCACTTGAATAAGGAGCACAACCTAGATGATAGATCAACGGCACAAGCTAGAGTGCAGATGCAAGTTGTCTC
GCAACTCGAACTTCAACTGCAGAAGGAGCGGGATCGACTACAAGCCATGATGCTACATCTCCACATGTCCAAGCAA
CAGAGTAACGTTGCTGCCATTGAACATGACAAGGATCCATCTAAAGCTCTATGTTTATCGAAGTTGAGTTCACCTCCA
CCTCCAGCTTACCTCACACAAGGTCAGTCTTGTCTATCAGCGAATAACTCGATAGCCCAAGGCGGTACCCCAGGTCCT
GGACCTGGACCTGGAACTGGACCTGGACCTGGGCCTGGACCATTGCCAGGTTCAGTCAGATCACCCGTGTCTTCCCTT
CGTCCATCAACACCTAGCCTCACCGTTCCTTCAAAACGAAGAATATGTGAGAAGTCAGCATTATCATTGGCTGGAGG
ATTACCATACATGTTGGAACGTGCTGGTTTGGATGTTCAACAGGAAATCCAAAGGAACCGTGAATTTTACAAGAATG
CTGATGTCAGACCTCCATTTACCTACGCTTCTCTTATCCGTCAGTCGATAATAGAGTCACCTGACAAACAGCTAACGC
TCAATGAAATCTACAACTGGTTTCAAAATACATTCTGCTACTTCAGAAGGAACGCAGCAACATGGAAGAATGCAGTG
CGCCACAACCTGTCTCTACACAAATGTTTCATGCGTGTGGAGAACGTGAAAGGCGCTGTGTGGACTGTGGACGAGGT
CGAGTTTTACAAACGGAGGCCACAGCGTTGCGTACCAGGGGATGCTCAATCCGAAGAAAGCTTATCATTGGGGAAA
AATCCGAGAGTTTTTGCAGACTCAATAATTGCCAACATTCAGGCACCGATGGGAGAGAATGCCTTTCCACAATACAT
CAGTACAACTCCTCCGCCACCACTACCTCCACCACTCAGGCCAGACAGTGGACCATCCTCACCTTCACCAAAAAAGC
TCAGGTTATCTCAAGAAGAGGAATTGCTCCGTCTTCAGCAAGAGAATGGGACCCATCCGTATCATCATCAGCAGCAG
CAACATCACCATCTTCAACAACAGGAGGAAAATGATCAAGAAGAAGTAGCTGAGGATTTGTCAATGGCACCTGAGG
AATCTCCAGCTGATCACAATGGAGCTGAATCGTAACAGTGGGGCGTCAGTTGAAATTTATGTAAAAACAACTTTTCT
ATCATATTAATACTCTGTAAATATTACACCTGTAAATAGATGGAGATCAAATTCCATCAGCTAAATAAATATTGTGAC
AACTCGGTAGG

>*NlFOXQ*¶

TGAAGGAGGAGAAGAAGGAGGAAAAGAAAAATAATAATAATGCTAGGCCAGCCTGGAGGAAATAATGGAGAGAA
CAACCAGACTGGTAGTCACCAACAGCCAAGTCCGACTGGGAGAGGTCCTCAAGCGCCCAGTCTTCCTATGTTGCAGC
CATTGGACCACTATCGGCTGCAGCTCTACAACTATGCAGTGCAAGCTGAGCGACTAAGATGTCAGCAGTTTATCAGTC
CTGGTGGAGCCGGGTCCCAATACCCAGGTACAGCCTCAGCTTTGTGTCCACCCTACCCGGCCACAGCAGCCTACCAT
CCCAGGCTGGCACTGTCAATGTCCCTCTTCCATCACCGCGTATTCCAGCCAGAGGAGCCCAAACCACAGTACAGCTA
CATTGGATTAATAGCAATGGCCATTCTATCATCCCCTGACCATAAACTGGTTCTCTCCGATATCTATCAGTATATTCTG
GACAACTATCCCTACTTCCGCACCCGGGGACCAGGTTGGAGGAACTCTATCCGTCACAACCTCTCTCTAAATGATTGT
TTTGTGAAAGCAGGAAGAAGTGCCAATGGAAAAGGACACTATTGGGCGATACATCCGGCAAATGTAGATGATTTCA
AGAAGGGGGACTTTAGGCGGAGGAAAGCGCAGCGAAAAGTGAGGAAACACATGGGTTTGGCTGTAGAGGATGATG
GAAATGACTCGAGCAGTCCACCACCTCCTCCATTGTCACCGACACCAGTTGGAACGATCTGGACTCCAGCGACGGTT
TTCCCCACAGCATTCCACCATCACCACCACCACCACCACCAAACCAACCATCACCACCATCATCACAACCATCACAC
AGATGTCAGCCTCACATCAGTCACTCTAAACAACATGGTTGGCCGAAAACGGCAGTTTGATGTTGCATCCTTACTGGC
TCCAGATGATGTTTCTGAGCAAATTTTACCTCTCCAGGAAAGAAAAATTACTGCGAAAGATCTTCCTATAAAAGGGG
AACCAAAGCTTGCACGATGCTTAGATGCTGAACAGAGCTTGGACATGGAATCCGCTCCTGAAGATGGGTCATCATCA
AAAAGGAAAAAAGTCTACAGCAGTGATGAAGATGCAGATGTGAGCAGTGAGGAAGTTGATGTGGTCTCTCACATTG
CTCCCAATGATAGCAATCGTCACCCTAGACCAACTCCCCTACATCACAGTATAGCATCTTCACCAGCTTGGCCCCTGC
CACTACCAGCAGTAACCTACCAGAACAGCTGTTTCAATCCCTCCATTGTTCCAGCACACATGGATCAGCATACAAAC
AGCTTGGTTGCTAGGTACTACCAGACTGCAGCCAGGCGATTGCAAATGCACAGACATCTGAACAATAGTAATAGTTT
TAGTGCTTACAATGATGATAATAATGGTAACGCGAATAGTAGTTGTAAGGATGATCAAGCAAGGGTGTCACCAACAG
CACTTGAAGAAGACAATGTTTGAAAAGAAAAGCTGGCATGAAATGTGTATGTCAACCGTGA¶

>*NlFoxT*

ACGCAAGTAAGAGTTCCAACGTGCTAGTTTTGATTTGATAGATGATTAGTTCAAACACCACACTACAGGAGAAGGAC
ACACTGCCGGCTGGTTGTGCATCATGATTGGACCCAATGTAATCACACTAGGAGGGAGTTGGCCCGAGTGTCAAACC
ACGGGTACTAAGGTCATCTCACAAAAACGGAGATGTGACGACGAGGGAACAGAGTGTCAAGATGGCGGCAGTGCA
GAAATGGTTTTGGTGAAGAAAAGACGCTACAAGAGGACATGTTCGGATGCTGAGAACAAGGCTCCTAAGGTCGGCA
GAGGAGTTGCTGTGGCTACAAAAACCATCAACAACAATCTGATTATCAATCAGGATAATGATCGGATCATCAGTGAT
GAAACAGTGTTGATCATCGATGCCAATTTTGGATGTACCAGAGCTTCCAGTGCTAGCAGCATCAGTCCAACCAATACT
GAAACCATCAGCAGTTTTTCTGACTCGATGCCATCATTGGAGGAAATCTACCCTGTCACCATTGGTGCAGAGTCTCAG
CCAATAAGGAGTGCCGCTCCTCAAACCGGAAGTGCTCCCATCCAGGCCGGAAGAGTATCTCCACCGCAACTAGAGG
CAGTTGACAATCCCAATCTGTCATGGCTACTCGACTTCAAGGTGTCATCACTGTTTGATCCCGATGGCAGTGAGGGCA
ATGTAGATGGATGCCTCCAAGGAGACGAGTGTCTACAGACGAGCTCTACGTCATCATCATCACCATCACCGCAATCA
TCATTGACAATGCCTCCTCCCCTCCCGCTGCCCGTGTCTCACCCCCCAACCAGCAATCGCCTCCTAAAACCGCCCTAC
ACCTACACAGAGCTCATAGAACAGGCACTCAACGAGAAGGGCCAACTCACAGTCTCCGGAATCTACACATGGATGT
CAGAGAAATATCCGTTTTTCAAAGCCCACGACGAACGATGGAAGAACTCGGTACGGCACAATCTGAGTATTAATCCA
CATTTTCGAAAAGGTATGAAGGCGTCTCAAGGATCCGGTCACTTTTGGACTTTGAGCACTCAAGGGGCTCCCCCTCCC
CCTCTTCATCCCAATCATGACACCTGGAAAATGACGGAAGTGAAAAGTGTGATTGAGACTGAGAGTGACGAGACTG
AGAAATCGTACATTGATGAGGTTGCCACGGCCACAGCCAGCATCGAGGAGCCTTCCTCTGGACTGGAGGACCCGTTC
TCACCCCCCCTCTCCATCACTATTCCTGCCGACAACTCGCCTGATCCCAGCCTAGCCAGAACTGCCGAAGAAATCCTG
TCAGGGGTCAAGAAAGAGGTCCAAGTGCAGTATCTTTCGGCCGATTCTGACTTTTTAAATCAAATGACGAGAGGCGA
ACTTGCAGATGAAACAAATCTAACAGAAGTTTCAACATCTTTTCTAATGAACGATTTGGGAACTGAAGTTATTTTGAA
TGACAGTTTTTTCTGTGACGATCTCAATTTCCAGTACTGCGAGCTCACTACTGCTCAGATGTGATCAGAATATCAGTCA
GGATTGAAGTTTTATTATGGAAATTCCTAGATATAGTTTTTGAATTGAGCAATCAAAGATGATGTGATGGAATTGATG
AGGA

Figure S1. The cDNA sequences of 18 BPH Fox genes were listed below. The initiators and terminators were boxed. The primers used for cloning, qRT-PCR, RNAi were highlighted in blue, red and yellow, respectively. The primers used for RNAi targeting the second non-overlapping region were highlighted in green. The sequences of FHD were underlined.

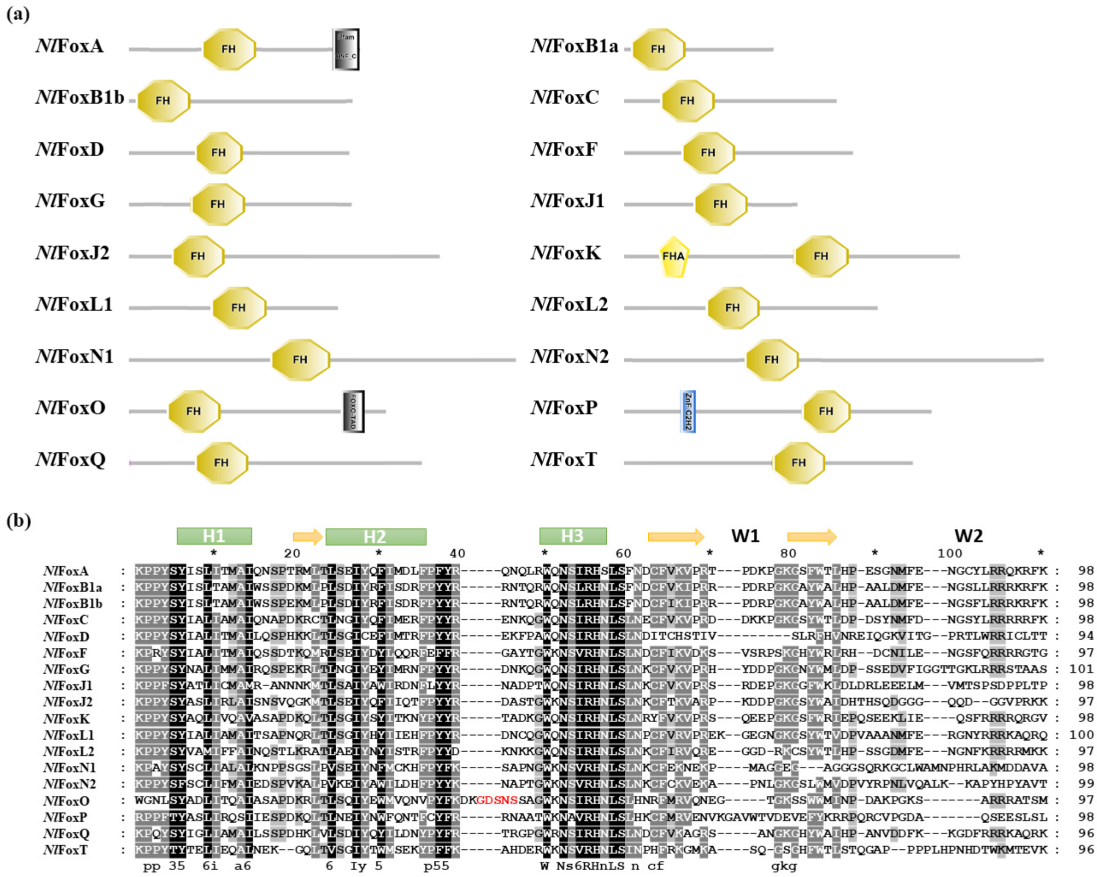

Figure S2. Sequence analyses of Forkhead genes in *N. lugen*. (a) Domains of the Fox proteins. FH, Forkhead domains; FHA, Forkhead-associated domain; HNF, hepatocyte nuclear factor; TAD, transactivation domain; Zn, zinc finger domain. (b) Alignment of amino acid sequences of FHD in BPH. Helices (H1–H3): green; β-strands: yellow; the first wing: W1; the second wing: W2; FoxO-specific sequence: red.

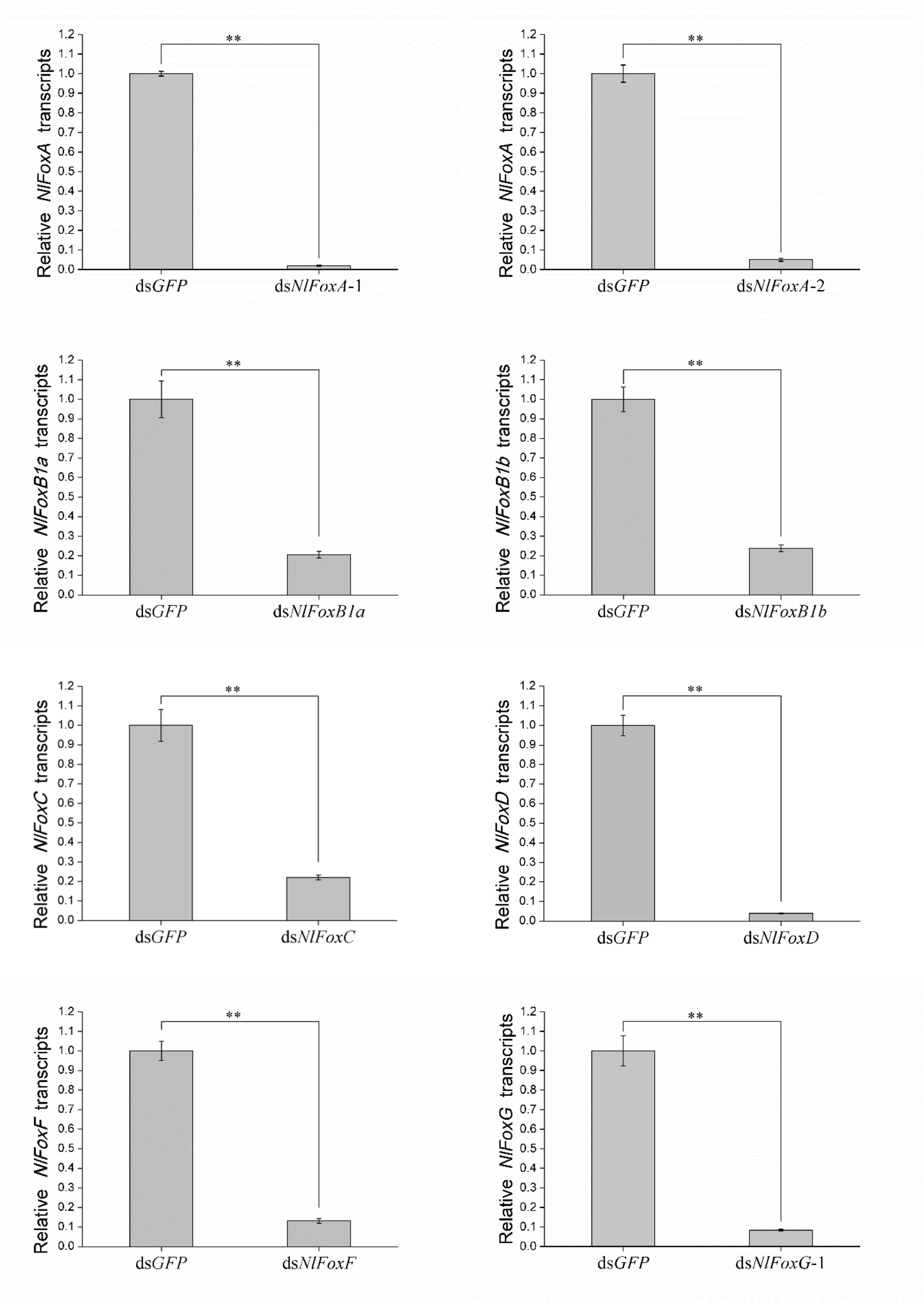

Relative NlFoxA transcripts
**
dsGFP
dsNlFoxA-1
Relative NlFoxA transcripts
**
dsGFP
dsNlFoxA-2
Relative NlFoxB1a transcripts
**
dsGFP
dsNlFoxB1a
Relative NlFoxB1b transcripts
**
dsGFP
dsNlFoxB1b
Relative NlFoxC transcripts
**
dsGFP
dsNlFoxC
Relative NlFoxD transcripts
**
dsGFP
dsNlFoxD
Relative NlFoxF transcripts
**
dsGFP
dsNlFoxF
Relative NlFoxG transcripts
**
dsGFP
dsNlFoxG-1
0.0
0.1
0.2
0.3
0.4
0.5
0.6
0.7
0.8
0.9
1.0
1.1
1.2

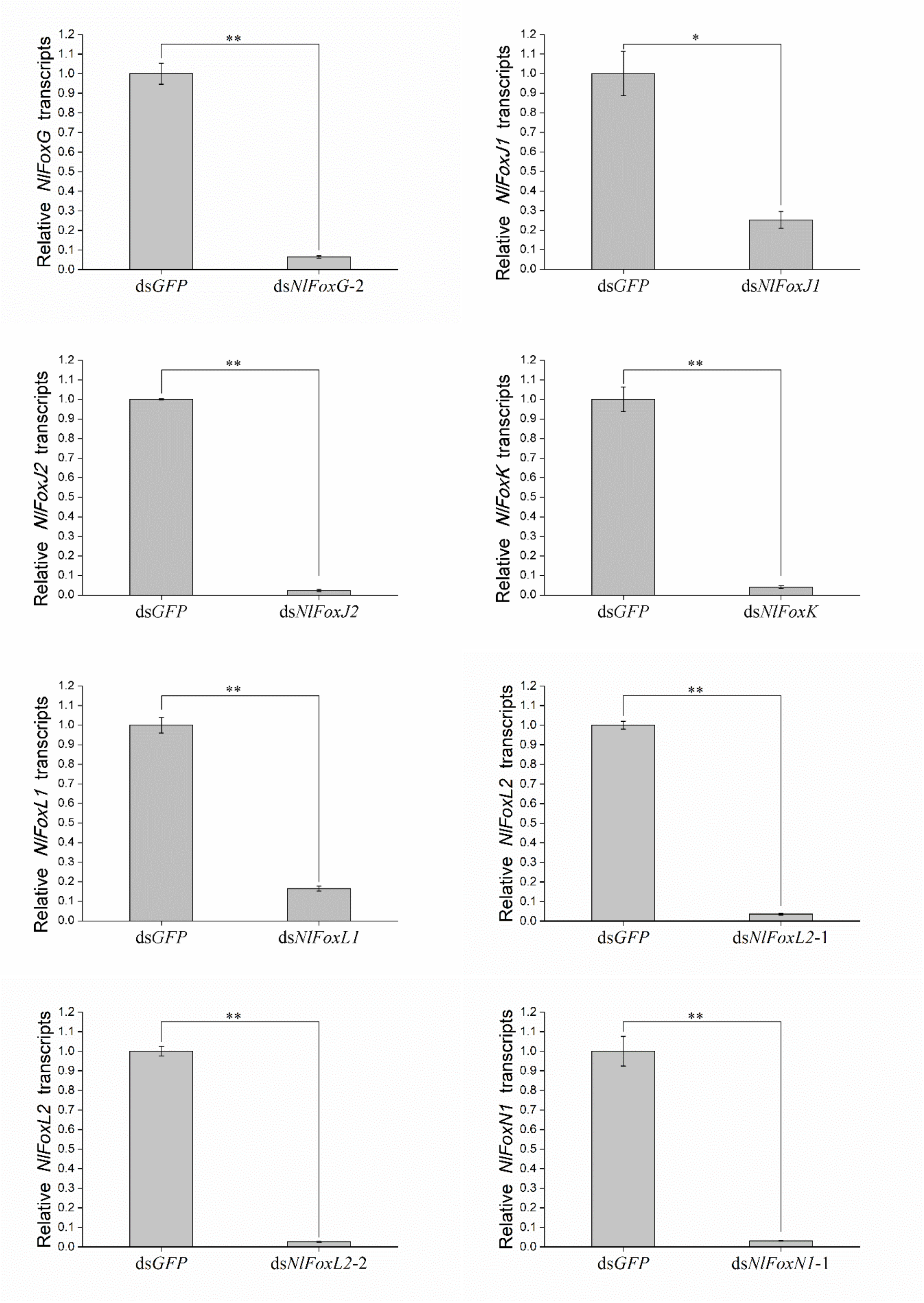
Relative NlFoxG transcripts
**
dsGFP
dsNlFoxG-2
Relative NlFoxJ1 transcripts
*
dsGFP
dsNlFoxJ1
Relative NlFoxJ2 transcripts
**
dsGFP
dsNlFoxJ2
Relative NlFoxK transcripts
**
dsGFP
dsNlFoxK
Relative NlFoxL1 transcripts
**
dsGFP
dsNlFoxL1
Relative NlFoxL2 transcripts
**
dsGFP
dsNlFoxL2-1
Relative NlFoxL2 transcripts
**
dsGFP
dsNlFoxL2-2
Relative NlFoxN1 transcripts
**
dsGFP
dsNlFoxN1-1
0.0
0.1
0.2
0.3
0.4
0.5
0.6
0.7
0.8
0.9
1.0
1.1
1.2

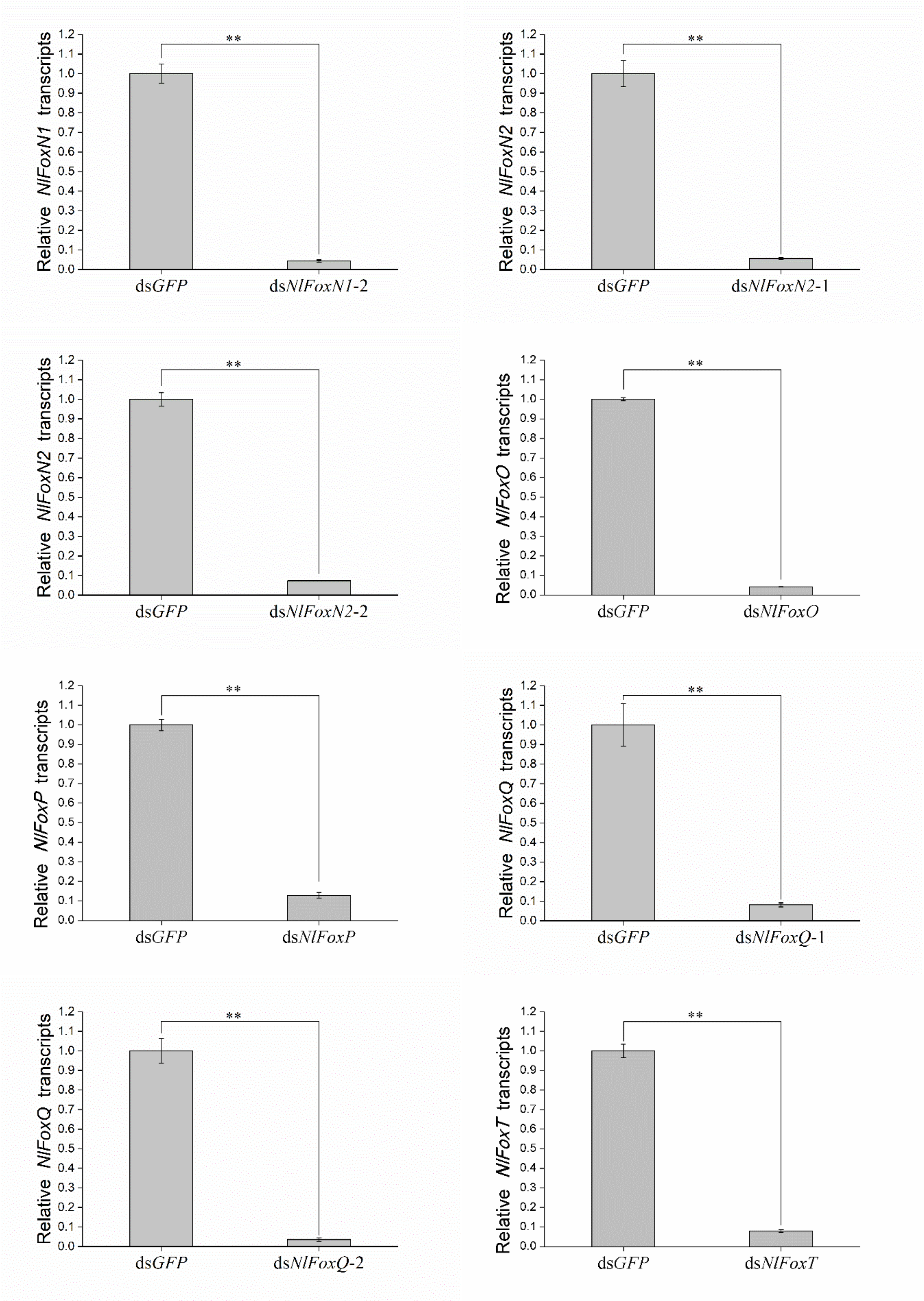
Relative NlFoxN1 transcripts
1.2
1.1
1.0
0.9
0.8
0.7
0.6
0.5
0.4
0.3
0.2
0.1
0.0
**
dsGFP
dsNlFoxN1-2
Relative NlFoxN2 transcripts
**
dsGFP
dsNlFoxN2-1
Relative NlFoxN2 transcripts
**
dsGFP
dsNlFoxN2-2
Relative NlFoxO transcripts
**
dsGFP
dsNlFoxO
Relative NlFoxP transcripts
**
dsGFP
dsNlFoxP
Relative NlFoxQ transcripts
**
dsGFP
dsNlFoxQ-1
Relative NlFoxQ transcripts
**
dsGFP
dsNlFoxQ-2
Relative NlFoxT transcripts
**
dsGFP
dsNlFoxT

Figure S3. RNAi efficiency by was determined by qRT-PCR. dsRNA (50 ng per insect; $n = 100$) was injected into BPHs at the very beginning of newly emerged adults. Total RNA samples for *NlFoxA*, *NlFoxJ2*, *NlFoxK*, *NlFoxN1*, *NlFoxN2* and *NlFoxO* were extracted from eggs within 2h after laid. Total RNA samples for *NlFoxD*, *NlFoxG* and *NlFoxQ* were extracted from eggs 48h after laid. Total RNA samples for *NlFoxB1a*, *NlFoxC*, *NlFoxF*, *NlFoxJ1* and *NlFoxL1* were extracted from eggs 72h after laid. Total RNA samples for *NlFoxB1b* and *NlFoxP* were extracted from eggs 96h after laid. Total RNA sample for *NlFoxL2* and *NlFoxT* were extracted from BPHs 3 days after injection. ds*GFP* was injected as negative control for the nonspecific effects of dsRNA. *Nl18S* was used as an internal control gene. Mean ± s.e.m. from three experiments. *$P < 0.05$, **$P < 0.01$ (Student's *t*-test), difference from ds*GFP*.

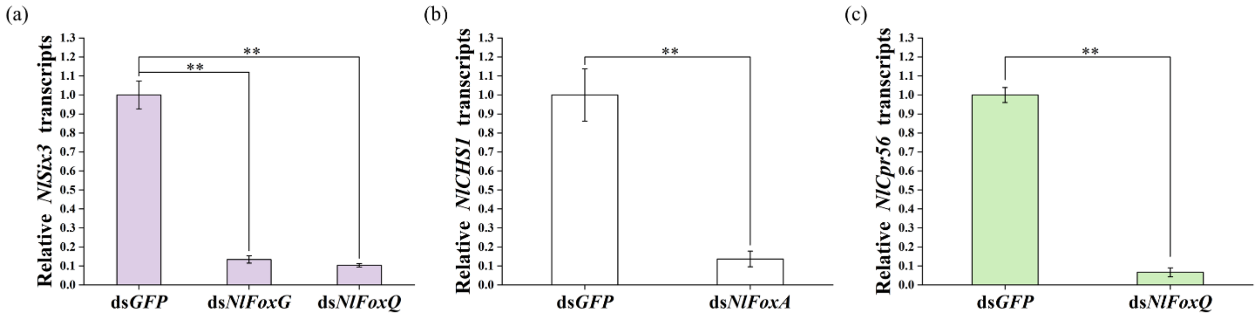

Figure S4. (a) Knockdown of *NlFoxG* or *NlFoxQ* both decreased expression levels of *NlSix3* in the eggs; (b) Knockdown of *NlFoxA* decreased expression levels of *NlCHS1* in the nymphs; (c) Knockdown of *NlFoxQ* decreased expression levels of cuticular protein genes in the nymphs. dsRNA for a (50 ng per insect; *n* = 100) was injected into newly emerged female adults (within two hours). Total RNA samples were extracted from eggs 48 h after laid. dsRNA for b-c (50 ng per insect; *n* = 100) was injected into BPHs at the very beginning of the 4th instar. Total RNA samples were extracted from BPHs 3 days after injection. ds*GFP* was injected as negative control for the nonspecific effects of dsRNA. Mean ± s.e.m. from three experiments. $^{**}P < 0.01$ (Student's *t*-test), difference from ds*GFP*.

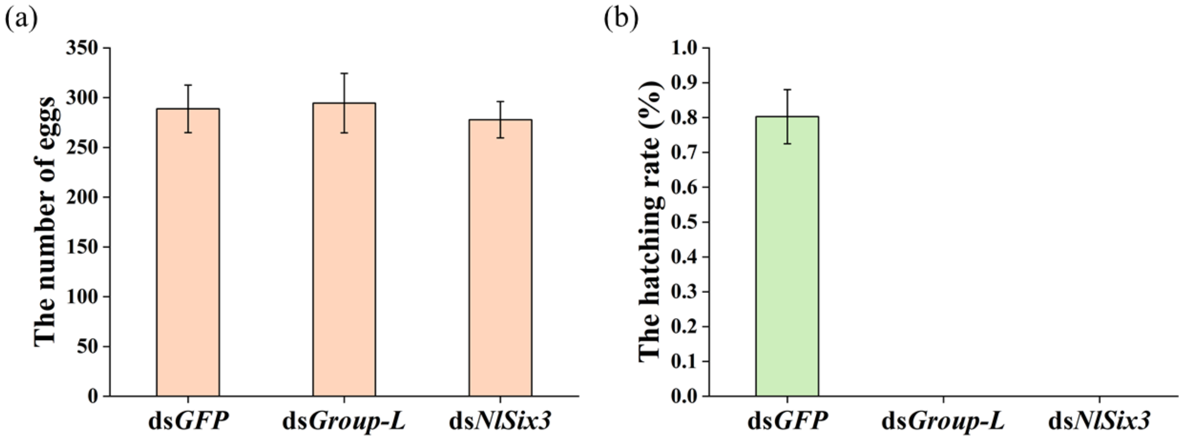

Figure S5. Knockdown of *Group-Late* or *NlSix3* prevented the hatchability. (a) The number of eggs; (b) The hatching rate. dsRNA (50 ng per insect; *n* = 100) was injected into newly emerged female adults (within two hours). ds*GFP* was injected as negative control for the nonspecific effects of dsRNA. Mean ± standard error of the mean (s.e.m.) from three experiments.

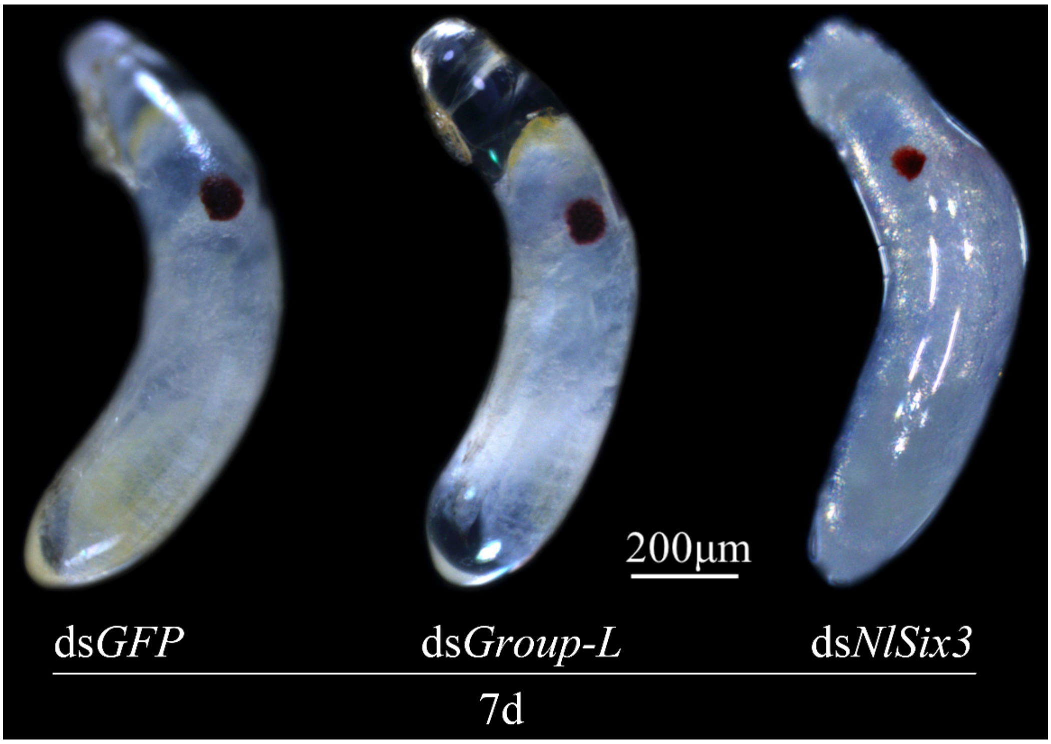

Figure S6. Lethal phenotypes of eggs injected with dsRNA for *Group-Late* or *NlSix3*. dsRNA (50 ng per insect; *n* = 100) was injected into newly emerged female adults (within two hours). ds*GFP* was injected as negative control for the non-specific effects of dsRNA.
